# Aging of α-Pinene Secondary Organic Aerosol by Hydroxyl Radicals in the Aqueous Phase: Kinetics and Products

**DOI:** 10.1021/acs.est.2c07630

**Published:** 2023-04-04

**Authors:** Bartłomiej Witkowski, Mohammed al-Sharafi, Kacper Błaziak, Tomasz Gierczak

**Affiliations:** Faculty of Chemistry, University of Warsaw, al. Żwirki i Wigury 101, 02-089 Warsaw, Poland

**Keywords:** hydroxyl radical, secondary organic aerosols, terpenoic acids, relative rate kinetics, cloud water, aging markers

## Abstract

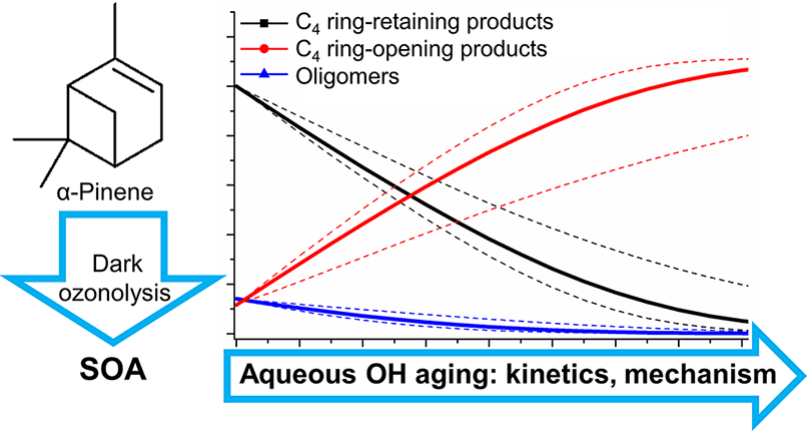

The reaction of hydroxyl radicals (OH) with a water-soluble fraction of the α-pinene secondary organic aerosol (SOA) was investigated using liquid chromatography coupled with negative electrospray ionization mass spectrometry. The SOA was generated by the dark ozonolysis of α-pinene, extracted into the water, and subjected to chemical aging by the OH. Bimolecular reaction rate coefficients (*k*_OH_) for the oxidation of terpenoic acids by the OH were measured using the relative rate method. The unaged SOA was dominated by the cyclobutyl-ring-retaining compounds, primarily *cis*-pinonic, *cis*-pinic, and hydroxy-pinonic acids. Aqueous oxidation by the OH resulted in the removal of early-stage products and dimers, including well-known oligomers with MW = 358 and 368 Da. Furthermore, a 2- to 5-fold increase in the concentration of cyclobutyl-ring-opening products was observed, including terpenylic and diaterpenylic acids and diaterpenylic acid acetate as well as some of the newly identified OH aging markers. At the same time, results obtained from the kinetic box model showed a high degree of SOA fragmentation following the reaction with the OH, which indicates that non-radical reactions occurring during the evaporation of water likely contribute to the high yields of terpenoic _aq_SOAs reported previously. The estimated atmospheric lifetimes showed that in clouds, terpenoic acids react with the OH exclusively in the aqueous phase. Aqueous OH aging of the α-pinene SOA results in a 10% increase of the average O/C ratio and a 3-fold decrease in the average *k*_OH_ value, which is likely to affect the cloud condensation nuclei activity of the _aq_SOA formed after the evaporation of water.

## Abstract 2

The kinetics and mechanism of aqueous OH aging of α-pinene SOAs were investigated in connection with the formation of aqueous SOAs in cloud water.

## 1 Introduction

Secondary organic aerosols (SOAs) are formed from isoprene and terpenes emitted primarily from biogenic sources.^1^ The oxidation of these atmospherically abundant, volatile organic compounds^2,3^ forms particulate matter (PM), which affects the climate and visibility and negatively impacts human health.^4,5^

Carboxylic acids are ubiquitous in the troposphere.^6,7^ C_1_–C_4_ acids contribute to the acidity of atmospheric aqueous particles, and their presence in cloud water often signifies the aqueous chemical aging of organic aerosols.^6−9^ Gas-phase oxidation of the most abundant monoterpene, α-pinene (α-P),^1^ generates large amounts of functionalized carboxylic acids, in addition to other oxygenated products.^10−13^ Multifunctional terpenoic acids (TAs) and acidic dimers contribute significantly to monoterpenoic SOAs;^14−18^ these compounds also play an important role in the SOA particle formation and growth.^16,19,20^ Furthermore, some poly(carboxylic acid)s, including 3-methyl-1,2,3-butanetricarboxylic (MBTCA) and hydroxydicarboxylic acids, were identified as important aging markers of terpenoic SOAs.^21−24^

Due to their high values of Henry’s law constants (10^7^–10^13^ M × atm^–1^),^25^ TAs and acidic dimers reside almost entirely in cloud and fog water.^26−28^ Therefore, such molecules are likely to undergo aqueous chemical aging, which can contribute to the formation of low-volatility organics in the atmosphere.^26,29−33^ Aqueous photochemical processing alters the molecular composition and physicochemical as well as optical properties of SOAs.^33−37^ Consequently, the formation and processing of SOAs in the aqueous phase have a direct impact on their cloud condensation nuclei and ice-nuclei activity,^38^ particle mass and number concentrations as well as the health effects of atmospheric PM.^39−42^ Because liquid water is a major tropospheric constituent,^39,43^ formations of aqueous SOAs (_aq_SOAs) are expected to significantly contribute to the global budget of organic aerosols.^44^ At the same time, the formation and processing of _aq_SOAs^43,45^ are still not well characterized,^43,46,47^ which limits our understating of the climate forcing of organic aerosols.

Aqueous processing (aging) of terpenoic precursors by the OH, which is the major atmospheric oxidant during the daytime,^5,46^ recently received attention as the potential source of _aq_SOAs.^26,29,33,48−54^ The kinetic data available to date strongly indicate that OH oxidation of TAs is likely to occur under realistic atmospheric conditions.^49,50,52,53^ Aqueous OH aging of the water-soluble fraction of α-P_SOA_ (α-P_SOA_aq__) yielded low-volatility, oxidized products^29,31,33^ but was also shown to decompose dimers.^33,48^

Atmospheric multiphase modeling of the formation and aging of SOAs requires detailed chemical mechanisms.^44,55,56^ However, a large number of studies published to date have been focused on single precursors,^26,27,29,31−33,57^ which limits the current understanding of the mechanisms of aqueous photochemical aging of terpenoic SOAs.

In this work, the aqueous oxidation of α-P_SOA_aq__ by the OH was investigated:
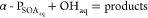
R1

This work presents a first attempt at an explicit parameterization of the aqueous processing of α-P_SOA_ by the OH using a kinetic model. The goals of this work included investigating the potential of the α-P_SOA_aq__ to undergo a reaction with the OH in atmospheric water-containing particles and studying the kinetics and products of R(I).

Here, an α-P SOA was generated in the flow-tube rector,^58^ collected on a filter, extracted into the water, and oxidized by the OH in the aqueous photoreactor.^54^ This work was focused on studying the chemical aging of the atmospherically abundant TAs and acidic oligomers contributing to α-P_SOA_aq__.^17,59,60^ For this reason, aliquots of the reaction solution were analyzed with liquid chromatography coupled with negative electrospray ionization mass spectrometry (LC-ESI(−)/MS).^14,17,59,60^ LC-ESI(−)/MS is capable of detecting as much as 72% of α-P_SOA_aq__.^14^ With the use of this hyphenated technique, dimers are also unambiguously distinguished from the lower-MW products, thereby providing detailed insight into the formation and evolution of individual TAs following R(I).

The bimolecular reaction rate coefficients for the oxidation of individual water soluble organic compounds (WSOCs) contributing to α-P_SOA_ by the OH (*k*_OH_, M^–1^ s^–1^) were measured using the relative rate method.^51^ The WSOCs under investigation were also analyzed with LC coupled with high-resolution MS to elucidate the structures of the detected tracers of α-P_SOA_.

## 2 Experimental Section

Materials and reagents are listed in Supporting Information (SI) Section S1. In all experiments, deionized water (18 MΩ × cm^–1^) was used.

### 2.1 Generation of α-Pinene SOA

Approximately 10 ppm of α-P and O_3_ were reacted in a 17 L flow-tube reactor under laminar flow conditions (5 min residence time).^58^ Relative humidity was 5%, and no OH scavenger was used. The SOA was collected on a 47 mm filter (EMFAB TX40H120-WW, Pall) for 2 h and kept at −85 °C before the aging experiments. Filters were extracted via mechanical agitation with 3 mL of ACN/H_2_O (1:1, v/v) for LC-ToF/MS measurements (Section 2.3) or with 70 mL of water for aqueous OH aging (Section 2.2) and kinetic measurements (Section 2.4).

### 2.2 Aqueous Oxidation by the OH

The solution of α-P_SOA_aq__ was transferred into a jacketed, Pyrex reaction flask with an internal volume of 60 mL; all experiments were carried out at 298 K in the aqueous photoreactor.^54^ The OH precursor (H_2_O_2_, concentration 10 mM) was photolyzed with four 9 W UVB lamps (PL-S 9 W/01/2P, Philips, peak emissions 310 nm). The reaction time was 60–80 min. The steady-state concentration of OH in the aqueous phase ([OH]_aq_) in the absence of organic reactants was approximately 6 × 10^–13^ M, as estimated by fitting the measured rate of H_2_O_2_ photolysis into a kinetic model.^61,62^ This value is within the range of [OH]_aq_ in atmospheric aqueous particles.^63,64^

### 2.3 Identification of α-Pinene Oxidation Products with Liquid Chromatography Coupled with High-Resolution Mass Spectrometry

The composition of the unaged SOA was studied with an LC20A liquid chromatograph coupled with an IT-TOF mass spectrometer (Shimadzu). The Waters Symmetry C18 column (150 mm × 2.1 mm, 3.5 μm, 100 Å), an eluent flow rate of 0.2 mL/min, ACN, and an aqueous solution of formic acid (0.03% v/v, pH = 2.8) were used. The gradient program was as follows: 0–2 min 5%B, 2–3 min increase to 12%B, 3–11 min 12%B, 11–12 min increase to 18%B, 12–28 min 18%B, 28–41 min increase to 95%B, 41–45 min 95%B, 45–46 increase to 5%B, and the total analysis time was 56 min. The ESI ion source was operating in the negative ionization mode, the spray voltage was −3.5 kV, the nebulizing gas flow was 1.5 mL/min, and the source temperature and desolvation line temperature were 200 °C. The detected [M-H]^−^ ions were assumed to be singly charged, deprotonated pseudo-molecular ions containing C, H, and O atoms. O/C values and double-bond equivalents were calculated with the acquired HR/MS data.

### 2.4 Kinetic Measurements Using Liquid Chromatography Coupled to Triple Quadrupole Mass Spectrometry

Aliquots of the reaction solution were periodically sampled from the photoreactor, quenched with catalase,^51^ filtered through 0.22 μm PTFE syringe filters, and injected into the Nexera X2 liquid chromatograph coupled with the LCMS-8040 triple quadrupole mass spectrometer (Shimadzu) equipped with an ESI ion source. The LC conditions were the same as those described in Section 2.3. The mass spectrometer operated at unit mass resolution; interface and desolvation line temperatures were 250 °C, and the heating block temperature was 350 °C. The ESI voltage was −5 kV, and nitrogen was used as the source and collision gas. Concentrations of analytes were monitored in the multiple reaction monitoring modes (Table S1).

Kinetic reference compounds, suberic, camphoric, azelaic, and sebacic acid,^51^ were added to the reaction solution before photooxidation. The pH of the reaction mixture was adjusted to 2 or 9 with HClO_4_ or NaOH to measure the *k*_OH_ values for the completely protonated and deprotonated TAs with p*K*_a_ values between 4 and 5.^65^

*k*_OH_ values for the α-P_SOA_aq__ constituents were derived using eq 1.
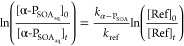
1

In eq 1, subscripts refer to the initial (0) and intermediate (*t*) concentrations of the individual constituents of α-P_SOA_aq__ and the reference compounds (ref), respectively. *k*_α-P_SOA__ and *k*_ref_ are the *k*_OH_ values for the compound under investigation and reference compounds, respectively. The rates of completely diffusion-controlled reactions (*k*_diff_) were estimated with the Smoluchowski equation (Section S3).^66^

### 2.5 Kinetic Box Model

In the kinetic box model, 8-hydroxy-pinonic, *cis*-pinonic, and *cis*-pinic acids were selected as the precursors for the rest of the TAs detected; pinonaldehyde and norpinonaldehyde, well-known products of α-P ozonolysis, were also included as precursors.^67,68^ All reactions in the model are listed in Table S6, and the initial concentrations of TAs under investigation in the reaction solution were between 1 and 150 μM (Table S7), as estimated with the standard solution of *cis*-pinonic acid using chromatographic peak areas for the TAs under investigation. These concentrations are comparable with the amounts of dissolved organic carbon observed in cloud water.^69^

In the box model, only the measured *k*_OH_ values for dimers were used, and the rate coefficients estimated with the structure–activity relationship (SAR, Section S5) at 298 K (*k*_OH_SAR__) were used for the rest of the TA under investigation. The *k*_OH_SAR__values listed in Table S2 were used for TAs because measured values for the lower-MW products were noticeably affected by their secondary formation from R(I). The yields of formation of the individual α-P_SOA_aq__ were obtained by fitting the model to the experimental data.

### 2.6 Control Experiments and Uncertainty

Each measurement was carried out a minimum of three times, and the uncertainties reported are propagated from the experimental uncertainties that were derived as 2σ values. Control experiments were carried out to verify if the decrease in the concentration of the TAs under investigation was only due to the reaction with the OH. When the photoreactor lamps were kept off, no reactions between α-P_SOA_aq__ and H_2_O_2_ were observed. Aliquots of the reaction solution were also stabilized by decomposing the leftover H_2_O_2_ (Section 2.4) to ensure that no further reaction occurred in the LC/MS autosampler rack (Figures S1 and S2). Ketoacids, including *cis*-pinonic acid, underwent slow photolysis induced by the 310 nm irradiation used in the photoreactor.^51^ The first-order disappearance rates (photolysis and hydrolysis) derived from the control experiments (dark and UV-only) were subtracted from the overall decay rates to obtain the *k*_OH_ values.^51^

## 3 Results and Discussion

### 3.1 Acids and Oligomers Detected in α-P_SOA_aq__

Structures for the detected α-P_SOA_aq__ were proposed based on the acquired HR-MS and MS/MS spectra (Section S4) and^70−73^ the literature data (Table 1).^10,16,18,74,75^ Hydroxy-pinonic acids were distinguished based on their retention order in the C_18_ stationary phase.^15^

**Table 1:**
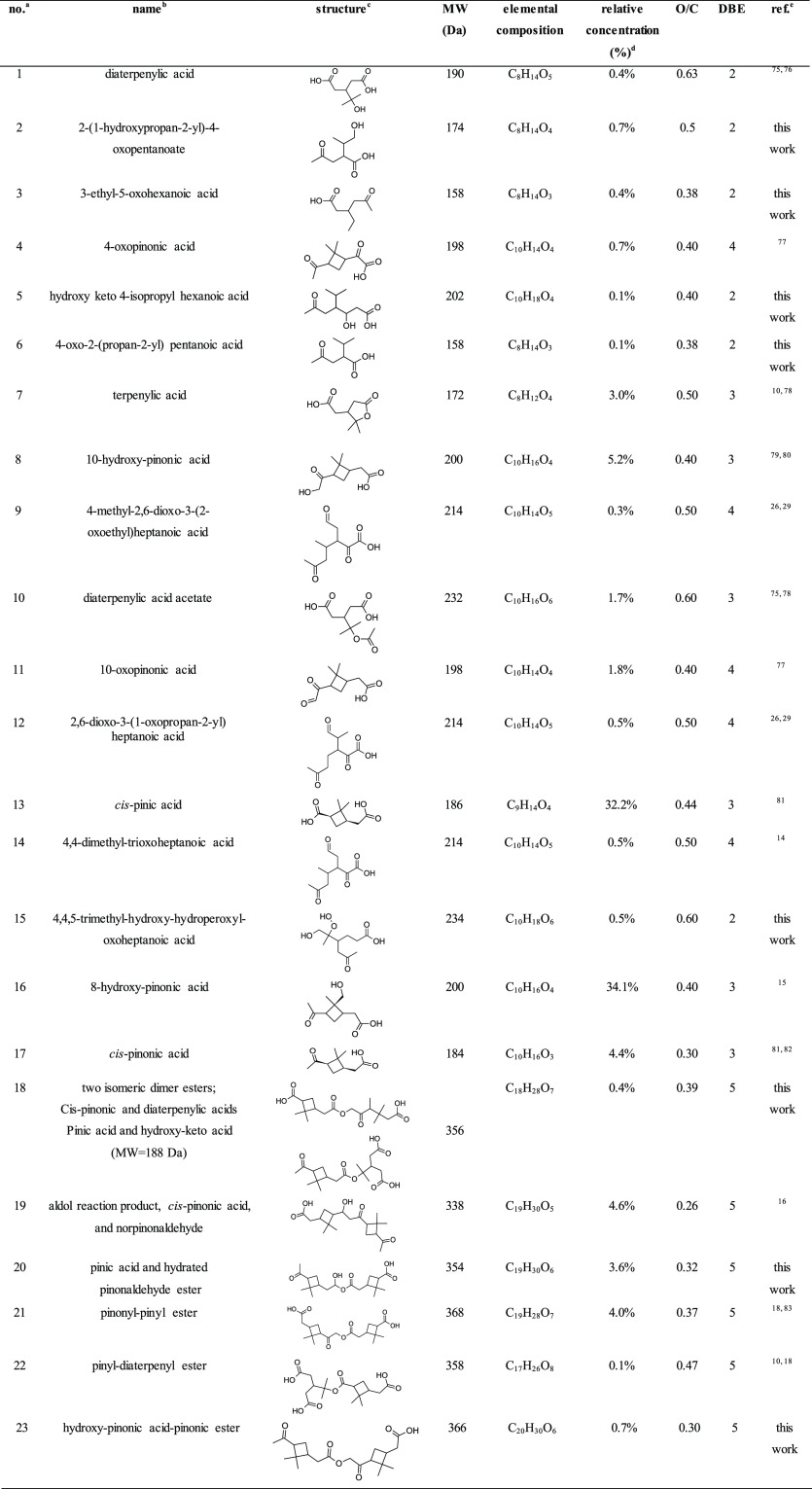
Water-Soluble Organic Constituents of the Unaged α-P_SOA_ Detected in This Work^74,76−82^

a Compounds are listed in their retention order (Table S1) on the C18 HPLC column used, roughly corresponding to the order of decreasing polarity and increasing hydrophobicity.

b For the compounds, tentatively identified in this work, substituent positions and only systematic names were given.

c Structural isomers could not be distinguished based on the acquired MS data.

d The value of the relative peak areas in the unaged SOA.

e The reference is listed only if the structure was proposed,^14^ based on the detailed interpretation of the acquired MS spectra.

Chromatographic peak areas were used to estimate the relative concentrations of individual TAs and dimer esters. α-P_SOA_ was dominated by early-stage products, *cis*-pinonic, *cis*-pinic, terpenylic, diaterpenylic, hydroxy, and oxo-pinic acids, diaterpenylic acid acetate as well as dimers with MWs 338, 358, and 368 Da, with some contribution from the C_4_-ring-opening compounds (Table 1),^29,31,57^ that were likely formed from the reaction with the OH due to the absence of the scavenger in the flow tube.

At the same time, the formation of some markers of the gas-phase OH aging of α-P_SOA_, including MBTCA and hydroxydicarboxylic acids,^21−24,75,83^ was not observed (see also Figure S3). Formation of MBTCA from the gas-phase oxidation of *cis*-pinonic acid and pinonaldehyde by the OH was reported.^21,22,72,84,85^ Furthermore, some studies tentatively identified MBTCA as one of the products of the aqueous OH oxidation of *cis*-pinonic^26,29,31^ and *cis*-pinic acids.^33^ However, MBTCA was not reported as the major product of the aqueous OH aging of α-P_SOA_,^49^ which is consistent with the results acquired in this work. Here, the absence of some of the common α-P_SOA_ OH aging markers may be due to their low formation yields in the aqueous solution or insufficient aging time since MBTCA was shown to be the higher-generation product of the OH oxidation of terpenoic precursors.^22,26,31^ Also, the OH aging in the bulk solution used in this work may not be an entirely adequate representation of the cloud-water processing of terpenoic SOAs,^57^ which can result in low formation yields for some of the aging products encountered in the ambient PM. There is a need to further investigate the formation of MBTCA and other typical gas-phase OH aging markers in the aqueous phase and at the air–water interface.

### 3.2 Kinetic Measurements

The *k*_OH_ values measured in this work were compared with the values predicted using the SAR (Section S5) at 298 K (Table 2) and with the literature data.^51,86,87^ In solution when every encounter between reactants leads to a reaction, the reaction rate is diffusion-limited.^88^ The *k*_diff_ values predicted (Section 2.4) that the rates of the individual α-P_SOAaq_ with the OH are below the diffusion-limited values; hence, they are only partially (up to 33%) controlled by diffusion (Table S2).

**Table 2:** Measured, SAR Predicted, and Literature *k*_OH_ Values for the Individual α-P_SOA_aq__and the Proposed Precursors for the Products of R(I)

name	*k*_OH_ (M^–1^ s^–1^) × 10^–9^ (298 K)^a^				proposed precursor	ref.
	this work(*RI*)^b^		SAR			
	pH = 2	pH = 9	pH = 2	pH = 9		
diaterpenylic acid	IN (2.3)		1.5	1.6	*cis*-pinic acid	(33)
2-(1-hydroxypropan-2-yl)-4-oxopentanoate	IN(1.4)		2.1	2.3	8-hydroxy-pinonic acid	
3-ethyl-5-oxohexanoic acid	IN(3.6)		2.2	2.4	*cis*-pinic acid	(33)
4-oxopinonic acid	1.3 ± 0.1	1.4 ± 0.2	2.6	2.6	*cis*-pinonic acid	(29,31)
hydroxy-keto 4-isopropyl hexanoic acid	IN(2.5)		2.7	2.9	*cis*-pinic acid	(33)
4-oxo-2-(propan-2-yl)pentanoic acid	IN(4.5)		2.2	2.4	*cis*-pinic acid	(33)
terpenylic acid	IN(2.7)		1.0	1.4	*cis*-pinic acid	(33)
10-hydroxy-pinonic acid	2.0 ± 0.3	2.6 ± 0.6	2.7	3.0	*cis*-pinonic acid	(29,31)
4-methyl-2,6-dioxo-3-(2-oxoethyl)heptanoic acid	IN(3.6)		1.0	1.1	8-hydroxy-pinonic acid	
diaterpenylic acid acetate	IN(2.0)		0.8	1.5	*cis*-pinonic acid	(29)
					*cis*-pinic acid	(33)
10-oxopinonic acid	1.0 ± 0.2	1.1 ± 0.1	1.7	2.0	*cis*-pinonic acid	(33)
2,6-dioxo-3-(1-oxopropan-2-yl)heptanoic acid	IN(3.8)		1.0	1.1	8-hydroxy-pinonic acid	
*cis*-pinic acid	1.7 ± 0.2	2.3 ± 0.2	2.2	2.9	norpinonaldehyde	(89)
4,4-dimethyl-trioxoheptanoic acid	IN(4.5)		2.9	3.4	8-hydroxy-pinonic acid	
4,4,5-trimethyl-hydroxy-hydroperoxyl-oxoheptanoic acid	3.1 ± 0.4	2.6 ± 0.3	2.9	3.4	*cis*-pinonic acid	(29)
					8-hydroxy-pinonic acid	
8-hydroxy-pinonic acid	1.9 ± 0.3	3.8 ± 0.4	3.6	3.9	*cis*-pinonic acid	(29)
*cis*-pinonic acid	2.1 ± 0.2	2.2 ± 0.2	2.7	2.8	pinonaldehyde	(89)
two isomeric dimer esters; *cis*-pinonic and diaterpenylic acids; pinic acid and hydroxy-keto acid (MW = 188 Da)	2.3 ± 0.2	2.3 ± 0.2	3.4	4.0		
aldol reaction product, *cis*-pinonic acid and norpinonaldehyde	4.3 ± 0.2	3.8 ± 0.5	4.9	5.3		
pinic acid and hydrated pinonaldehyde ester	4.4 ± 0.5	3.9 ± 0.1	4.7	4.7		
pinonyl-pinyl ester	3.8 ± 0.3	3.7 ± 0.1	4.1	4.5		
pinyl-diaterpenyl ester	5.9 ± 0.5	4.4 ± 0.3	3.0	3.7		
hydroxy-pinonic acid-pinonic ester	4.0 ± 0.7	3.7 ± 0.4	5.0	5.4		

a IN indicates the increase in the concentration.

b Relative increase following R(I) after the first-generation products and dimers were almost completely removed.

The *k*_OH_ values measured in this work under acidic and basic conditions for *cis*-pinic, *cis*-pinonic, and 4-oxopinonic acids are generally in good agreement with the previously reported data (Table 2). However, the *k*_OH_ values measured here for some TAs were lower than the values obtained using pure standards.^49,51^ The estimated *k*_SAR_ values (Table S5) were also noticeably higher than the *k*_OH_ values measured in this work (Table 2).^49,51^ Therefore, *k*_OH_ values measured here for lower-MW products are likely affected by their secondary formation in the reaction solution.^29,31−33,89^ Previously, only the formation of terpenylic acid (*m*/*z* 171) was observed from reaction 1 under very similar conditions.^49^ Note, however, that the authors did not compare their results with SAR predictions and used slightly outdated kinetic data; their study was focused on a lower number of TAs,^49^ which might explain these discrepancies. Nevertheless, the kinetic data presented here (Tables 2 and S5) show that the OH reactivity of TAs under investigation is within the range 10^8^–10^10^ M^–1^ s^–1^ for the organics encountered in the atmosphere.^46^

### 3.3 Aqueous OH Aging of α-P_SOAaq_

To better understand the mechanism of aging upon reactions with OH, a box model was set up. Estimated *k*_SAR_ values (Table S5), measured *k*_OH_, and literature data compiled in our previous study^51^ all showed little pH dependence of OH reactivity of the TAs under investigation. Average *k*_SAR_ values were used in the box model for lower-MW products because the measured values were affected by the formation of the TAs under investigation from R(I) (Figure S15).

The concentrations of α-P_SOA_aq__ increased during R(I) (Table 2). Because 8-hydroxy-pinonic, *cis*-pinonic, and *cis*-pinic acids were the major components of α-P_SOA_aq__, they were selected as the precursors of the OH aging markers (Section 2.5). The precursors for the rest of the TAs under investigation were assigned based on the HR-MS measurements (Table 1) and the data available in the literature (Table 2).^29,31,33,89^ A relatively good agreement between the experimental data and modeling results was obtained for the early-stage products of α-P oxidation (Figure S16).

**Figure 1:**
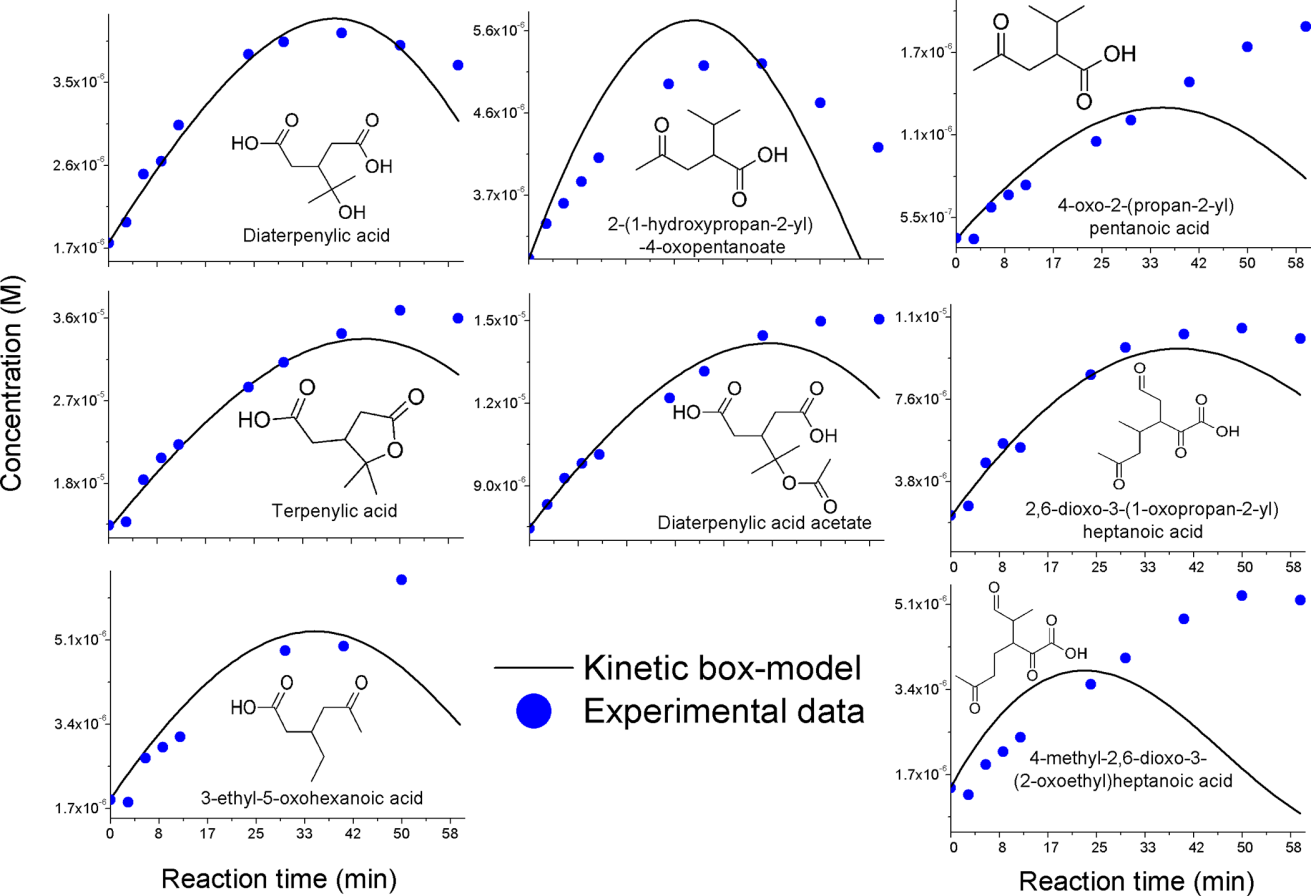
Experimental and modeled temporal concentration profiles of aqueous OH aging markers of the α-PSOA.

An initial increase of concentrations of C_4_-ring-opening products (Table 1) was observed during R(I), including terpenylic acid and its derivatives,^33,49^ which is accurately reproduced by the kinetic model (Figure 1). Furthermore, to the best of our knowledge, this is the first observation of the formation of diaterpenylic acid and diaterpenylic acid acetate from R(I) in an aqueous solution. The modeled yields of higher-generation products from *cis*-pinonic, *cis*-pinic, and 8-hydroxy-pinonic acids ranged from 1–2 to 25–35% with higher values corresponding to the formation of terpenylic acid (Figure S18). The model yields of terpenylic acid are unrealistically high, which points out the relevance of the OH reaction with the non-acidic, terpenoic precursors;^74^ similar conclusions can be presented about the modeled yields of *cis*-pinonic and *cis*-pinic acids from pinonaldehyde and norpinonaldehyde (Figure S18).

Modeling results also revealed that a significant fraction of the products of R(I) is not represented in the model (Figure S18 and Table S6). Therefore, a large portion of α-P_SOA_aq__ can be volatilized and concerted into lower-MW and^31,90^ non-acidic products that are not quantified under the LC/MS analysis conditions used in this work. C_2_–C_4_ acids are practically not retained by the C_18_ column used, and their ionization efficiency in ESI is likely low. These results imply that a large fraction of alkoxy radicals (RO) formed from R(I) likely undergoes the β-scission reaction, yielding lower-MW products,^91^ like oxalic acid, which is abundant in cloud water.^8^

In the box model, oligomers are decomposed by the OH (Figure S17),^48,49^ which is consistent with the acquired experimental data. At the same time, the formation of pinyl-diaterpenyl ester (MW = 358 Da) was observed from the OH aging of α-P_SOA_ under dry (RH < 1%) conditions;^75^ a radical-mediated mechanism is most likely involved in the formation of this dimer ester.^18,75^ Therefore, the formation of some acidic dimers from the reaction of TAs with the OH likely involves interfacial reactions of RO and peroxy (RO_2_) radicals.^75,92^ An increase in the concentration for some of the functionalized derivatives of *cis*-pinic and *cis*-pinonic acids, including compounds with MW 158 Da, was reported following gas-phase OH aging of α-P_SOA_,^75^ whereas in this work, the concentrations of C_4_-ring-retaining products decreased during R(I) (Figure S16). Such a result likely reflects the different composition of α-P_SOA_ in the gas and aqueous phases. α-P_SOA_aq__ is enriched in highly soluble TAs, whereas in the gas phase, higher amounts of more volatile carbonyls (aldehydes) were observed.^67,68^ These carbonyls are likely precursors of the C_4_-ring-retaining TAs, resulting in an observed increase in their concentration following gas-phase OH aging of α-P_SOA_.^75,93^

Analysis of specific dimers (this work), as well as previously reported results of non-targeted analyses, revealed that oligomers either are not formed or are decomposed following the aqueous OH reaction with terpenoic SOAs at cloud-relevant and also at higher concentrations of the precursors.^33,48,49,53^ At the same time, the formation of higher-MW products was observed from the aqueous OH oxidation of C_2_–C_4_ precursors carried out in the bulk solution.^61,94,95^ However, in our previous work, increasing the concentration of *cis*-pinonic acid to 10 mM still did not result in the formation of higher-MW products during the aqueous oxidation by the OH.^29^ Evidently, very high concentrations of the TAs are required for oligomer formation, which may occur in the atmosphere during evaporation of droplets. These conclusions are supported by the reported formation of oligomers in aerosols composed of saturated diacids.^92^ Likely that at cloud-relevant concentrations used in this work, alkyl radicals will react primarily with molecular oxygen, suppressing the formation of higher-MW products in aqueous solutions.^61,95^

The dimers detected here also underwent slow hydrolysis, under acidic and basic conditions, which was accompanied by a noticeable increase in the concentrations of terpenylic acid (Figures S1 and S2). However, within the time scale of the photooxidation experiments, hydrolysis was a minor process; therefore, dark hydrolysis of dimer esters is unlikely to compete with the OH-mediated reactions under the realistic atmospheric conditions.^92^

## 4 Environmental Implications

The atmospheric lifetimes of TAs under investigation due to the reaction with the OH were calculated with eq 2.^88^
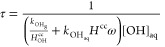
2

In eq 2, τ is the total lifetime for a given compound due to the reaction with the OH in both gas and aqueous phases, ω is the liquid water content (LWC) (m^3^/m^3^), *k*_OH_g__ and *k*_OH_aq__ are the *k*_OH_ values in the gas and the aqueous phase at 298 K, respectively, and *H*_OH_^cc^ and *H*^cc^ are the dimensionless Henry’s law constants for the OH (764)^96^ and TAs (Table S8), respectively. [OH]_aq_ is the cloud and fog water concentration of OH (M),^46,97^ which is connected with the [OH]_gas_ via Henry’s law equilibrium (eq 2).^88^ The *k*_OH_g__ values were estimated with SAR,^98,99^ and the *H*^cc^ values were estimated with HenryWin 4.11 (Table S8).^100^

The six TAs, identified as the major components of α-P_SOA_aq__ (Table 1), will undergo oxidation by the OH primarily in the aqueous phase when the LWC is ≥1 × 10^–3^ (g/m^3^) (Figure 3).^26,29,101^ Furthermore, dimers are expected to react in the aqueous phase, even in aerosols and haze with the LWC as low as 1 × 10^–6^ (g/m^3^) (Figure S19). These results indicate that in clouds with the LWC between 0.01 and 1 (g/m^3^), the aqueous OH aging of α-P_SOA_ will occur almost exclusively in the aqueous phase. At the same time, the OH aging of α-P_SOA_ under dry conditions is more likely to yield new oligomers, which are classified as extremely low-volatility compounds^14^ and are expected to exist exclusively in the particle phase.^19^

Functionalization of TAs under investigation following R(I) is also unlikely to contribute to an increase in acidity via the formation of acids with lower p*K*_a_ values (Table S10).^65,102,103^ Instead, aging of SOAs may enhance the acidity of aqueous particles via the formation of lower-MW acids (e.g., oxalic and formic acids) from R(I).^9^

The results obtained in this work showed a high degree of fragmentation of TAs and rapid decomposition of acidic dimers; oligomers present in SOAs are classified as extremely low-volatility compounds.^14,104^ These results argue against an efficient formation of _aq_SOAs from R(I). Conversely, very high mass yields of _aq_SOAs from the OH reaction with terpenoic precursors (40–60%) were reported.^26,26^ As previously reported, evaporation of nebulized aqueous solutions of TAs did not result in the dark formation of _aq_SOAs.^26^ It is therefore likely that functionalized, non-acidic products of R(I) undergo non-radical accretion reactions^105^ during droplet evaporation. These dark reactions can contribute to the formation of _aq_SOAs from terpenoic precursors, like oligomer formation from carbonyls,^106^ that are formed from RO_2_ radicals via Russell or Bennett–Summers mechanisms.^107^ The reported yields of TAs from the α-P + O_3_ reaction are as high as 40%;^108^ therefore, the formation of terpenoic _aq_SOAs from R(I) is likely to occur in the atmosphere.

**Figure 2:**
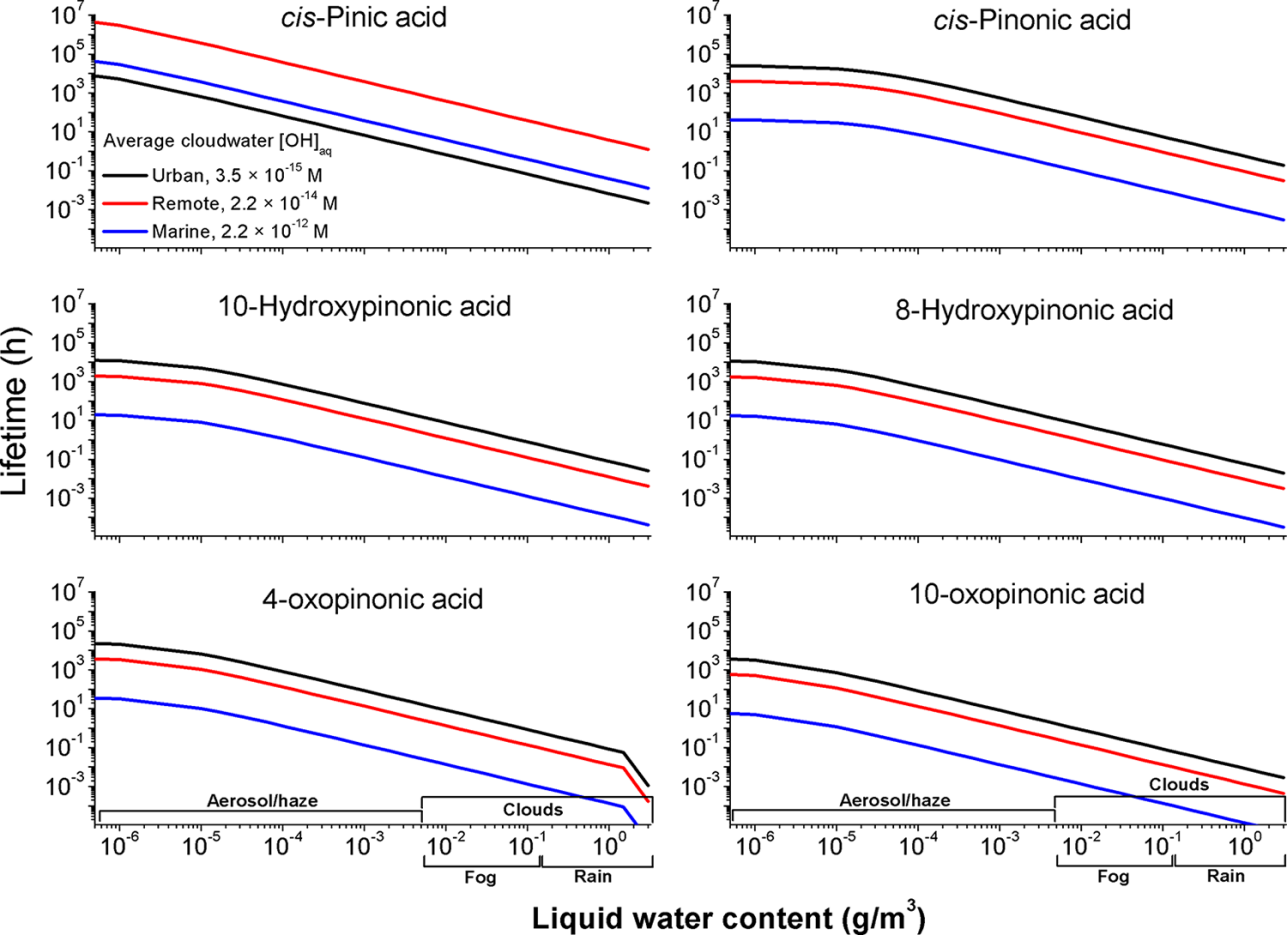
Estimated total lifetimes of the TAs (early-stage products) due to the reaction with the OH in the gas and aqueous phases. The shorter, combined lifetime, decreasing with the increasing values of the LWC, shows that a given precursor undergoes aqueous processing under realistic atmospheric conditions. Colors represent average [OH]_aq_ in different clouds.

The lifetimes estimated for the early-stage products (Figure 2) and dimers (Figure S19) corresponded to the in-cloud processing time between 2.5 weeks and 2 min, depending on the cloud-water concentration of OH.^64^ These results strongly indicate that R(I) can efficiently compete with other removal mechanisms, like photolysis or wet deposition,^27^ even after taking into account the fact that clouds are present about 15% of the time.^26,49^

Very recently, a large, light-driven formation of OH was reported in cloud droplets, resulting in the [OH]_aq_ as high as 3.5 μM,^109^ which is six orders of magnitude higher as compared with the upper limit [OH]_aq_ in marine clouds (Figure 2).^64^ Following this burst of OH_aq_, the gas-phase OH aging of α-P_SOA_ may become practically irrelevant in clouds, thereby further enhancing the cloud-water formation of terpenoic _aq_SOAs.

**Figure 3:**
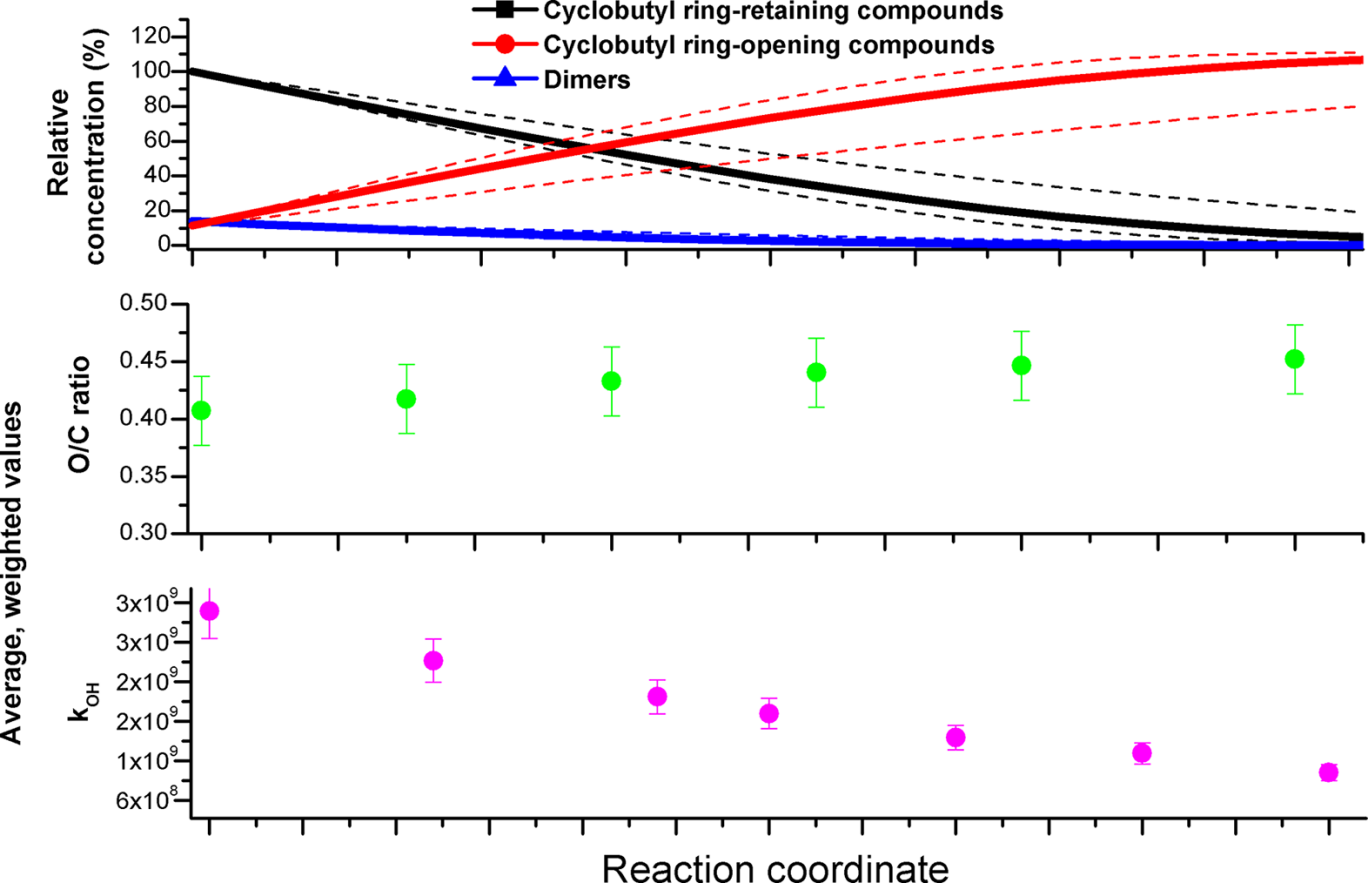
Changes of α-P_SOA_aq__composition and properties during aqueous aging by the OH. Average, weighted values were obtained by multiplying the O/C and *k*_SAR_ values of the individual TAs by their relative concentrations during R(I).

First-generation products and dimers are removed following aging by the OH, which results in about a 10% increase in the average O/C ratio of TAs contributing to α-P_SOA_aq__. Moreover, a 3-fold decrease in the overall OH reactivity of α-P_SOA_aq__ was observed (Figure 3), which can be attributed to the formation of more oxidized, lower-MW products (Figure S14), which significantly lowers the OH-scavenging ability of the aged _aq_SOA. The results presented in Figure 3 also underline the possibility of a simplified representation of the in-cloud formation of _aq_SOAs from R(I).^44,49,110^ The concentrations of the C_4_-ring-opening products, including newly identified OH aging markers as well as terpenylic acid and related compounds, increased several-fold during R(I);^26,31,32,57^ these compounds, however, are not exclusively formed in the aqueous phase.^75^ The kinetic data acquired in this work indicate that saturated oligomers are more reactive toward OH as compared with the majority of the lower-MW products (Figures S14 and S15).

## Supporting Information Available

List of materials and reagents, results of control experiments, results of HR-MS measurements and MS/MS analysis conditions, derivations of *k*_diff_ and *k*_SAR_ values and comparison with the experimental data, MS^2^ spectra and proposed fragmentation pathway for some α-P_SOA_aq__, details of the kinetic box model, *k*_OH_ (gas and aqueous) values and *H*^cc^ values for all α-P_SOA_aq__, and measured and estimated p*K*_a_ values for all TAs investigated (PDF)
