# Supplementary material for: Aging of α-Pinene Secondary Organic Aerosol by Hydroxyl Radicals in the Aqueous Phase: Kinetics and Products
Source: Environ Sci Technol. 2023 Apr 4;57(15):6040–51. doi: 10.1021/acs.est.2c07630 (PMC10116591; doi:10.1021/acs.est.2c07630)
Supplement: Supplementary file 1 — es2c07630_si_001.pdf [file es2c07630_si_001.pdf]

Supporting Information

Aging of α-pinene secondary organic aerosol by hydroxyl radicals in the aqueous phase; kinetics, and products

Bartłomiej Witkowski,[1*] Mohammed al-Sharafi,[1] Kacper Błaziak,[1] and Tomasz Gierczak[1]

[1]University of Warsaw, Faculty of Chemistry, al. Żwirki i Wigury 101, 02-089 Warsaw, Poland

Summary: 42 pages, 10 tables, 19 figures

S1. Materials and reagents

Catalase from bovine liver (2000-5000 units/mg protein), hydrogen peroxide solution in water (≥30%, ultratrace, no stabilizers added), α-pinene (≥98.0%), perchloric acid (ACS reagent grade, 70% solution in water), *cis*-pinonic acid (98%), *cis*-pinic acid (≥98.5%), suberic acid (≥99.5%), camphoric acid (≥98.5%), azelaic acid (≥98.0%) and sebacic acid (≥99.0%) as well as HPLC-grade solvents and LC/MS-grade eluent additives: acetonitrile, formic acid, were purchased from Sigma – Aldrich (Germany). Sodium hydroxide(≥98.8%) was purchased from Avantor Performance Materials (Gliwice, Poland). Deionized (DI) water (18 MΩ cm^{-1}) was used as LC eluent and to prepare the reaction solutions were prepared using Direct - Q3 Ultrapure Water System (Millipore). Synthetic zero-air (≤ 3 ppm of H_2O and ≤0.1 ppm of hydrocarbons), and UHP oxygen (≥99.999 %) that were used in the flow reactor were supplied by Multax (Stare Babice, Poland).

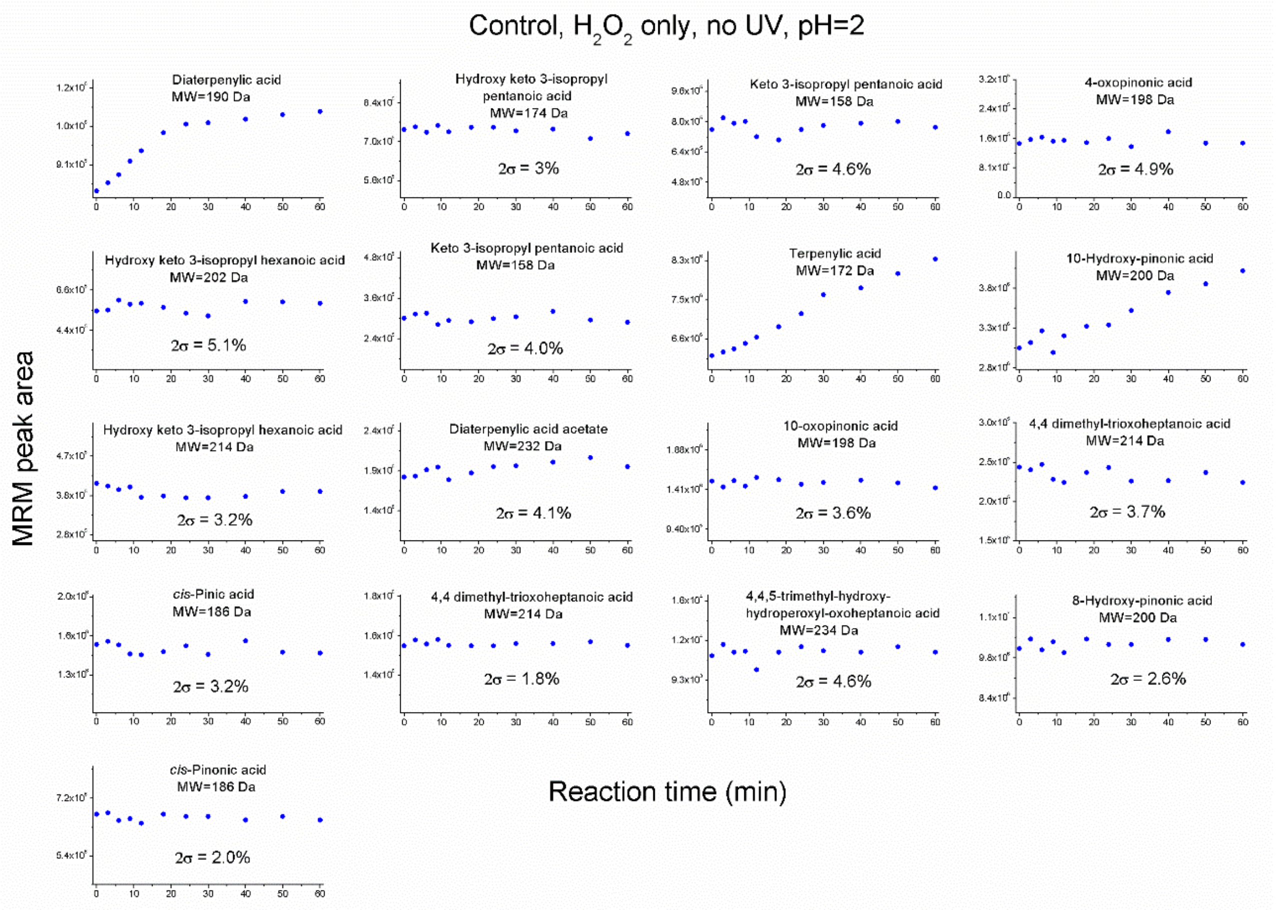

Figure S1 Temporal concentration profiles obtained for the lower-MW products from the dark control experiments, pH=2. For stable compounds, 2σ values (two values of the relative standard deviation) were calculated from all data points.

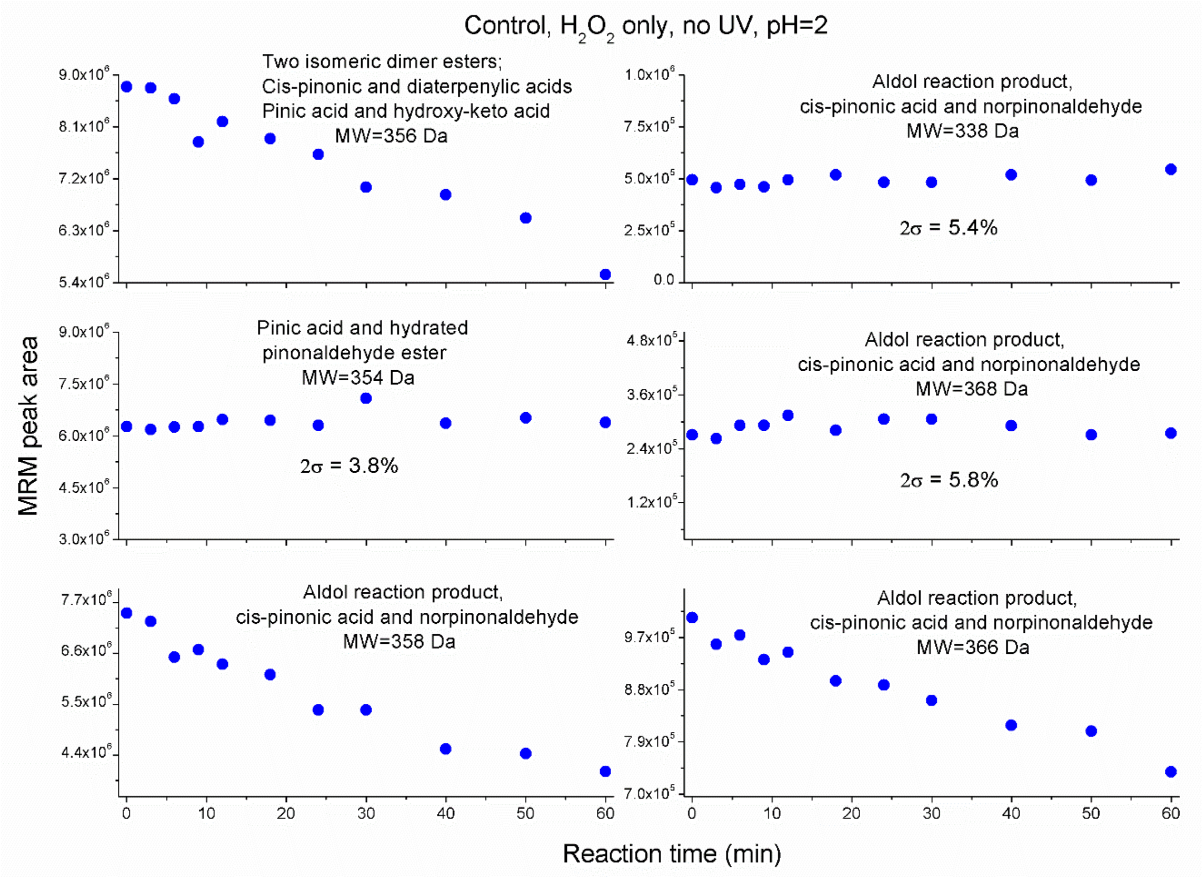

Figure S2 Temporal concentration profiles obtained for oligomers from the dark control experiments, pH=2. For stable compounds, 2σ values (two values of the relative standard deviation) were calculated from all data points.

Results presented in Figs. S1 and S2 confirmed that H_2O_2 wasn't reactive towards SOA under acidic pH conditions, within the time scale of the measurement. Almost identical results were obtained when the dark control experiments were carried out under basic pH conditions.

The data summarized in Figs. S1 and S2 underline that the samples were efficiently stabilized after taking them out of the reactor since no changes in their concentrations (corresponding to the MRM peak areas) were observed. Furthermore, exponential concentration profiles obtained (Figs. S16-S17) also strongly indicate that the samples were not reacting any further in the autosampler rack, because the disappearance rates are a function of reaction time and do not depend on the time that the samples wait in the HPLC instrument prior the injection. This conclusion was further confirmed by analyzing every sample twice (double injection into the instrument).

Only slow (note the Y-axis scale in Figs. S1 and S2, acidic, and basic hydrolysis were observed for some dimers in the dark control experiments, which was additionally confirmed carrying out control experiments without H_2O_2, as detailed in section 2.6.

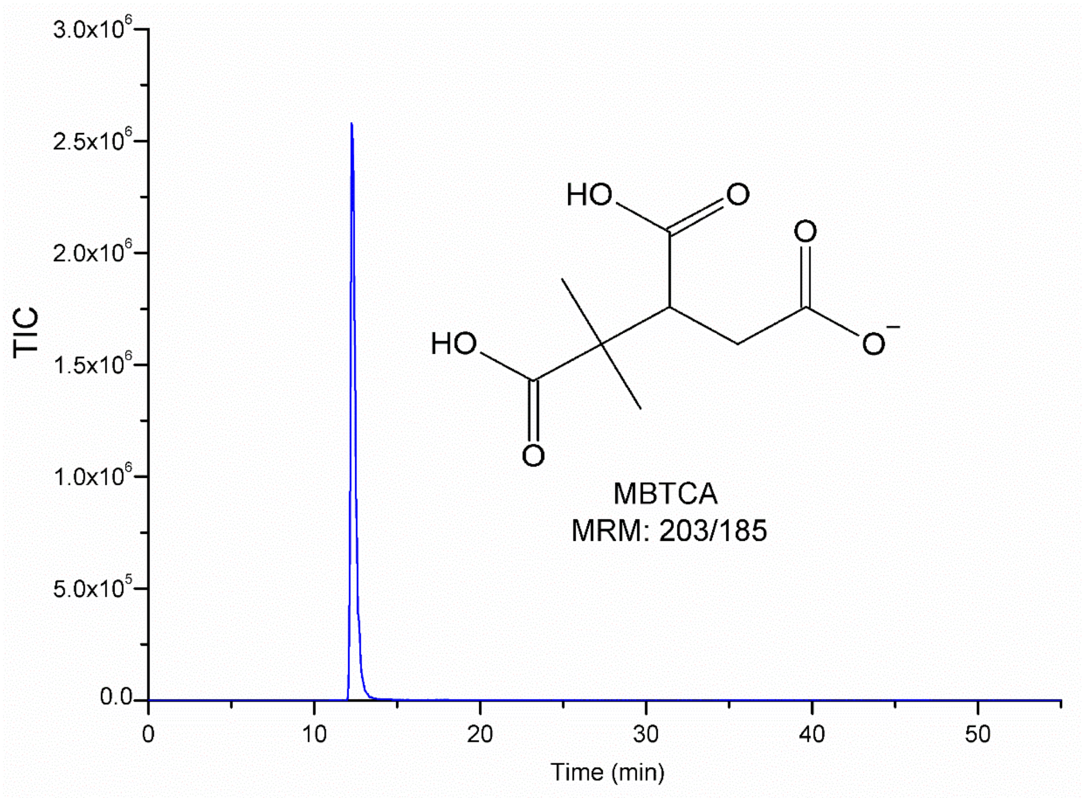

Figure S3 MRM chromatogram of the authentic standard of MBTCA acquired using the triple-quadrupole instrument (section 2.4 in the main text)

LC/MS analysis of the authentic standard of MBTCA confirmed that under the experimental conditions used, it was possible to detect this compound in SOA samples. The analyte was not eluted in the void peak and minimal tailing of the chromatographic peak corresponding to MBTCA was obtained. Therefore, it would be possible to observe the formation of significant quantities of these compounds in the initial measurements that were carried out in a scan mode with both ToF and triple-quadrupole instruments.

S2. Ions monitored in the MRM and results of the HR-MS measurements

Table S1 Results of the HR-MS measurements and MRM analysis conditions of the water-soluble fraction of α-P SOA

LC-ToF-MS (identification)					LC-MS/MS (kinetic measurements)[b,c]			
**Name**	**Retention time (min)**	**Measured m/z**	**Δ m/z[a]**	**Elemental composition**	**Q1/Q3 (m/z)**	**Q1 prebias (V)**	**CE (arb)**	**Q3 prebias (V)**
Diaterpenylic acid	7.5	189.0821	0.00525	$C_8H_{14}O_5$	189/85	23	19	16
2-(1-hydroxypropan-2-yl)-4-oxopentanoate	8.0	173.0835	0.00177	$C_8H_{14}O_4$	173/115	21	10	21
Adipic acid	8.4	Kinetic reference compound			145/101<br>145/83	20<br>19	30<br>29	19<br>19
3-ethyl-5-oxohexanoic acid	10.0	157.0872	0.00018	$C_8H_{14}O_3$	157/113	30	12	29
4-oxopinonic acid	10.1	197.0864	0.00447	$C_{10}H_{14}O_4$	197/153	24	9	28
Hydroxy keto 4-isopropyl hexanoic acid	10.2	201.1051	0.00081	$C_{10}H_{18}O_4$	201/143	25	10	25
4-oxo-2-(propan-2-yl) pentanoic acid	10.6	157.0896	0.00258	$C_8H_{14}O_3$	157/113	29	11	20
Terpenylic acid	12.0	171.0691	0.00282	$C_8H_{12}O_4$	171/127	20	12	23
Hydroxy-pinonic acid	12.6	199.0996	0.00202	$C_{10}H_{16}O_4$	199/181	24	11	18
4-methyl-2,6-dioxo-3-(2-oxoethyl)heptanoic acid	12.1	213.0745	0.00235	$C_{10}H_{14}O_4$	213/141	25	13	25
Pimelic acid	13.6	Kinetic reference compound			159/97<br>159/141	20<br>20	18<br>19	21<br>21
Diaterpenylic acid acetate	18.5	231.0922	0.00588	$C_{10}H_{16}O_6$	231/171	27	11	17
10-oxopinonic acid	17.0	197.0825	0.00057	$C_{10}H_{14}O_4$	197/153	24	9	28
2,6-dioxo-3-(1-oxopropan-2-yl) heptanoic acid	16.3	213.0737	0.00273	$C_{10}H_{14}O_5$	213/141	25	5	22

Table S1, continued…

LC-ToF-MS (identification)					LC-MS/MS (kinetic measurements)[b,c]			
**Name**	**Retention time (min)**	**Measured m/z**	**Δ m/z[a]**	**Elemental composition**	**Q1/Q3 (m/z)**	**Q1 prebias (V)**	**CE (arb)**	**Q3 prebias (V)**
Suberic acid	19.7	Kinetic reference compound			173/111	21	20	19
					173/83	21	21	19
cis-Pinic acid	16.8	185.083	0.00107	$C_9H_{14}O_4$	185/141	22	15	27
2,6-dioxo-3-(1-oxopropan-2-yl) heptanoic acid	19.8	213.0778	0.00095	$C_{10}H_{14}O_5$	213/141	25	13	25
4,4,5-trimethyl-hydroxy-hydroperoxyl-oxoheptanoic acid	19.9	233.1034	0.00034	$C_{10}H_{18}O_6$	233/183	28	11	18
8-Hydroxy-pinonic acid	24.5	199.0995	0.00301	$C_{10}H_{16}O_4$	199/181	24	11	18
Camphoric acid	23.8	Kinetic reference compound			199/155	24	23	17
cis-Pinonic acid	25.3	183.1058	0.00313	$C_{10}H_{16}O_3$	183/139	22	13	28
Azelaic acid	32.3	Kinetic reference compounds			187/125	20	22	20
Sebacic acid	35.2				201/183	25	24	19
Two isomeric dimer esters;<br>Cis-pinonic and diaterpenylic acids<br>Pinic acid and hydroxy-keto acid (MW=188 Da)	35.5	355.1786	0.00237	$C_{18}H_{28}O_7$	355/185	28	16	19
Aldol reaction product, cis-pinonic acid, and norpinonaldehyde	36.2	337.2043	0.00225	$C_{19}H_{30}O_5$	337/319	26	16	22
Pinic acid and hydrated pinonaldehyde ester	36.2	353.1961	0.00086	$C_{19}H_{30}O_6$	353/185	26	19	19
Pinonyl-pinyl ester	36.4	367.1808	0.00457	$C_{19}H_{28}O_7$	367/185	28	20	19
Pinyl-diaterpenyl ester	37.2	357.1545	0.00099	$C_{17}H_{26}O_8$	357/185	27	17	19
Hydroxy-pinonic acid-pinonic ester	37.8	365.196	0.00096	$C_{20}H_{30}O_6$	365/199	29	17	21

[a]The difference between the measured exact mass and monoisotopic formula weight [b]MRM conditions were optimized by directly injecting SOA filter extract into the ion source of the triple-quadrupole mass spectrometer (section 2.4); only the most intense fragmentation ions were included in the final method [c] Each MRM was monitored only in a small-time window, centered around the retention time of the analyte.

S3. Derivation of the k_{diff} values

The method for calculating the rates of the completely diffusion-controlled reactions of the individual α-P_{SOA} with the OH in the aqueous phase (k_{diff}, $M^{-1}s^{-1}$) via Smoluchowski equation was described in detail in the previously published studies. [1-3]

Briefly, the group-contribution method was used to estimate the critical volumes (V_c, cm^3) that were subsequently used to obtain the molar volumes (V_m) for each compound listed in Table 1 in the main text.[4] The V_m values obtained were used to calculate the radii (r, cm^{-1}) for the compounds listed in Table 2. The r values derived were then used to calculate diffusivities using the modified Strokes-Einstein equation.[5] Finally, the r and D (cm^2 s^{-1}) values were used to calculate the k_{diff} via the Smoluchowski equation – S1.

$$k_{diff} = 4 \cdot 10^{-3} \cdot \pi \cdot \mathrm{N_A} \cdot (\mathrm{r_{OH}} + \mathrm{r_{acid}}) \cdot (\mathrm{D_{OH}} + \mathrm{D_{acid}}) \qquad \text{(SI)}$$

The k_{diff} values are estimated with eq. SI are listed in Table S2.

Table S2 The r, D and k_{diff} values and k_{OH} values estimated with SAR at 298 K

No.	r(cm)×10[8]	r_{OH}(cm) ×10[8]	D (cm^2 s^{-1}) ×10[6]	D_{OH} (cm^2 s^{-1}) ×10[5]	k_{diff}($M^{-1}s^{-1}$) ×10[-9]	k_{OH} (M^{-1} s^{-1}) ×10[-9] (SAR)[a,]		Diffusion contribution[b]
						pH=2	*pH=9*	
1	4.3		6.6		14.5	1.5	1.6	19%
2	4.2		6.8		14.5	2.1	2.3	16%
3	4.2		6.8		14.5	2.2	2.4	8%
4	4.2		6.8		14.5	2.6	2.6	20%
5	4.5		6.0		14.8	2.7	2.9	7%
6	4.2		6.8		14.5	2.2	2.4	8%
7	4.0		7.6		14.3	1.0	1.4	13%
8	4.3		6.5		14.6	2.7	3.0	7%
9	4.5		6.0		14.7	1.0	1.1	17%
10	4.6		5.8		14.8	0.8	1.5	22%
11	4.4		6.2		14.6	1.7	2.0	21%
12	4.5	2.2	6.0	2.3	14.7	1.0	1.1	26%
13	4.2		6.8		14.5	2.2	2.9	19%
14	4.5		6.0		14.7	2.9	3.4	24%
15	4.6		5.9		14.8	2.9	3.4	33%
16	4.3		6.5		14.6	3.6	3.9	30%
17	4.3		6.5		14.6	2.7	2.8	28%
18	5.3		4.5		15.6	3.4	4.0	22%
19	5.2		4.7		15.5	4.9	5.3	33%
20	5.3		4.5		15.6	4.7	4.7	19%
21	5.3		4.5		15.6	4.1	4.5	16%
22	5.3		4.5		15.6	3.0	3.7	8%
23	5.3		4.5		15.6	5.0	5.4	20%

[a]*Neighboring parameters for hydroxyl moieties were used for the oxygen atoms embedded in non-aromatic rings (terpenylic acid) and for the hydroperoxyl groups (compound 15), esters were treated as carboxylic acids ,*[b]*Average value for pH=2 and 9 is given*

S4 Identification of terpenoic acids contributing to $\alpha - P_{SOA_{aq}}$ based on the acquired HR-MS and MS/MS spectra

Firstly, fragmentation spectra of the commercially available, functionalized carboxylic acids were acquired; results are summarized in Table S3.

Table S3 Fragmentation patterns of model carboxylic acids

Name	Group	Observed neutral losses from the $[M-H]^-$ ions[a]									
		18 Da	*44 Da*	*46 Da*	*48 Da*	*58 Da*	*60 Da*	*62 Da*	*88 Da*	*90 Da*	*105 Da*
Glutaric acid	Dicarboxylic acids	2	3	0	0	0	0	2	0	0	0
Adipic acid		2	3	0	0	0	0	3	0	0	0
Pimelic acid		2	2	0	0	0	0	3	0	0	0
Suberic acid		2	1	0	0	0	0	3	0	2	0
Azelaic acid		2	1	0	0	0	0	3	0	2	0
Sebacic acid		2	1	0	0	0	0	2	0	0	0
cis-Pinic acid		2	2	0	0	0	0	2	0	0	0
Tricarballylic acid	Tricarboxylic acids	3	2	0	0	0	0	2	2	0	0
3-methyl-1,2,3-butanetricarboxylic acid (MBTCA)		3	1	0	0	0	0	2	2	1	3
1,2,4-Butanetricarboxylic acid		3	2	0	0	0	0	2	2	2	2
Tartaric acid	Hydroxycarboxylic acids	1	1	3	0	0	0	3	0	0	2
Malic acid		3	2	1	0	0	0	3	0	0	0
8-Hydroxyoctanoic		1	1	3	2	0	0	1	0	0	0
2-Hydroxycaproic acid		1	0	3	1	0	0	0	0	0	0

[a]Intensity scale for the corresponding fragment ion: 1 (observer), 2 (intermediate), 3 (major)

Table S3 Fragmentation patterns of model carboxylic acids, continued…

Name	Group	Neutral losses from the [M-H]⁻ ions[a]									
		18 Da	44 Da	46 Da	48 Da	58 Da	60 Da	62 Da	88 Da	90 Da	105 Da
Levunilic acid	Keto acids	1	2	0	0	0	0	0	0	0	0
4-Acetylbutyric acid[b]		1	0	0	0	1	0	0	0	0	0
2-Ketoglutaric acid		2	3	1	0	1	0	3	2	0	0
cis-Pinonic acid		1	3	0	0	1	1	0	0	0	0
6-Oxoheptanoic		2	2	1	0	1	2	2	0	0	0
7-Oxooctanoic		2	2	0	0	1	2	2	0	0	0

[a]Intensity scale for the corresponding fragment ion: 1 (observer), 2 (intermediate), 3 (major)

As previously reported, almost all of the deprotonated pseudo-molecular ions $[M-H]^-$ of C_5-C_{10} carboxylic acids listed in Table S3 exhibited neutral losses of H_2O, CO_2 as well as combined neutral loss of water and decarboxylation. [6, 7] For all carboxylic acids under investigation neutral loss of water and decarboxylation (loss of CO_2) was observed. Interestingly, loss of 88 Da (double decarboxylation) was not observed for dicarboxylic acids and was only observed for the tricarboxylic acids as well as 2-ketoglutaric acid.[8] Hence, for the elimination of two carbonyl groups, a third, likely charge bearing oxygen atom seems to be necessary.

Furthermore, some ketoacids exhibited characteristic neutral loss of 58 Da via charge-remote McLafferty-like rearrangement.[8, 9] For the negatively charged ions formed in ESI, six-membered transitions state are preferred, but fragmentation mechanisms involving four-membered transition states were also observed. [10] However, the presence of characteristic ketoacids fragments dependent on the molecular structures of the ketoacids under investigation; was only observed for the compounds with more than five carbon atoms; the formation of specific fragment ions depending on the length of the carbon backbone was observed previously for the negatively charged ions of carboxylic acids in ESI/MS ion sources. [6] For instance, short-chain ketoacids did not form these fragment ions, likely because the formation of a five-membered transition state in the case of levunilic acid would not be favorable. Accordingly to the mechanism of McLafferty rearrangement , elimination of 58 Da from ketoacids is only possible when the acetone moiety is attached to a secondary (but not tertiary) carbon atom.[8]

Under these assumptions, structural elucidation of detected $\alpha - \mathrm{P}_{SOA_{aq}}$ (see Table 1 in the main text) was carried out. Fragmentation spectra of the new tracers of $\alpha - P_{SOA}$ identified in this work are presented in Fig. S4.

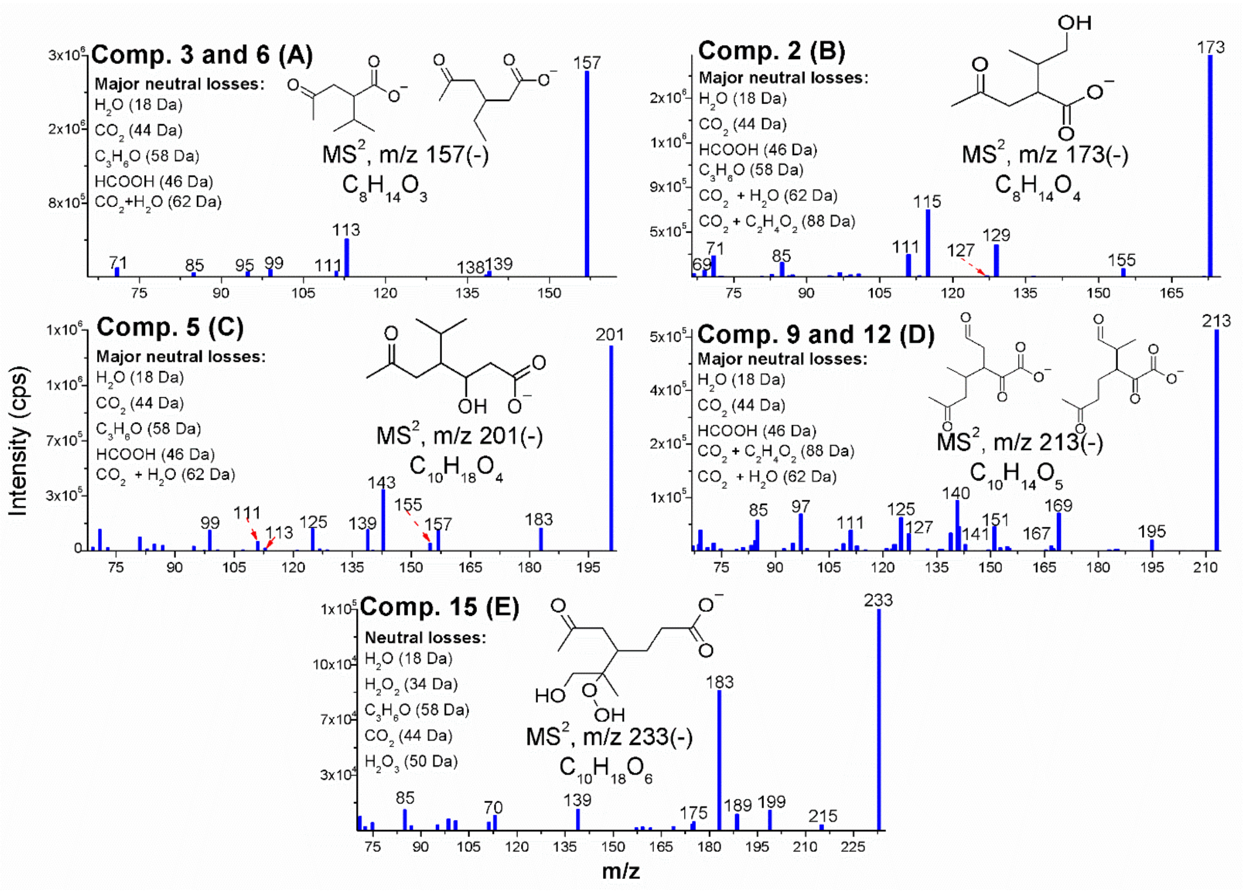

Figure S4 Fragmentation spectra for (A) compounds 3 and 6, (m/z 157), (B) compound 2, (m/z 173), (C) compound 5 (m/z 201), (D) compounds 9 and 12, (m/z 213) and (E) compound 15, (m/z 233)

Proposed fragmentation mechanisms for the compounds tentatively identified in this work (Fig. S4) are presented in Fig. S5-S9.

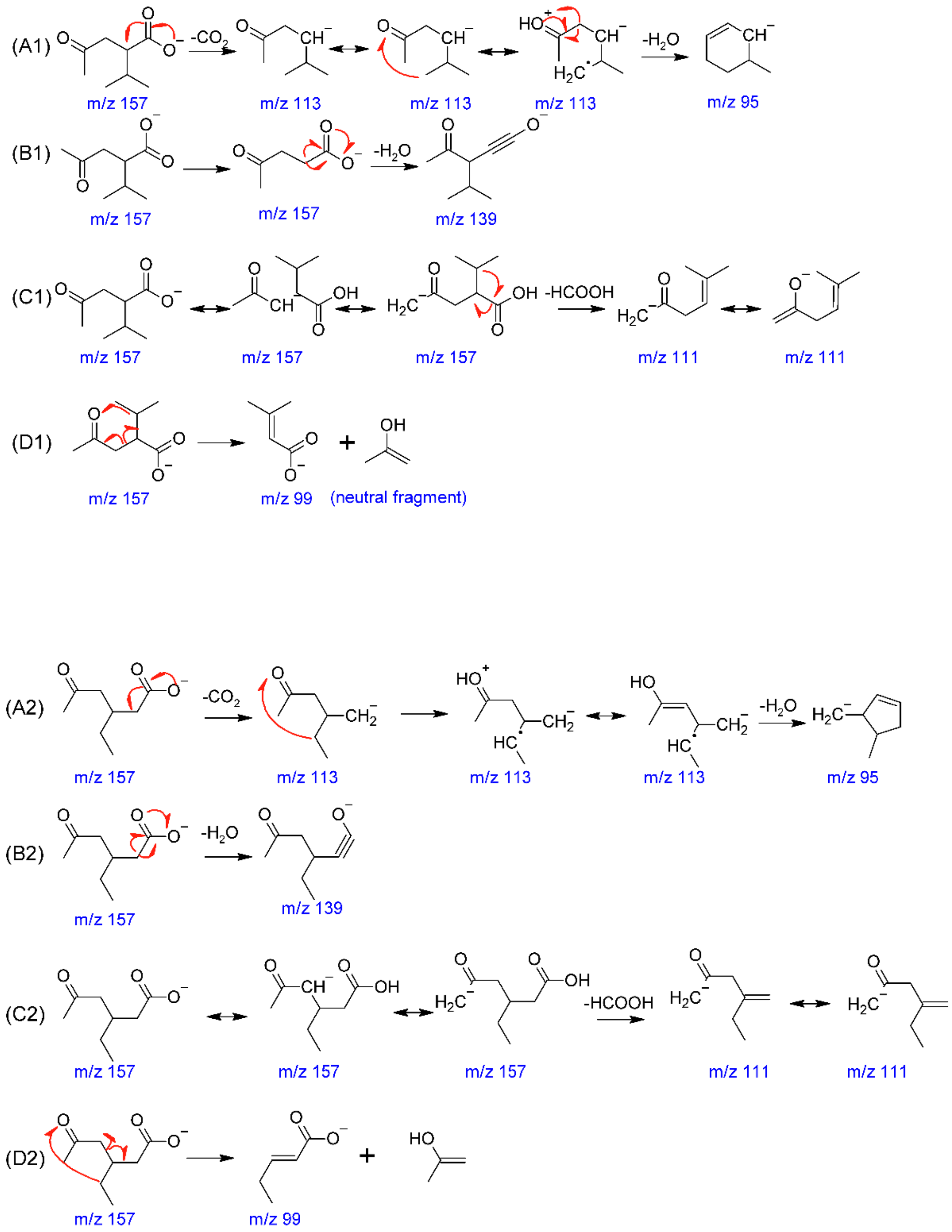

Figure S5 Proposed fragmentation mechanism of the two isomeric compounds with MW=158 Da

Two isomeric products detected as m/z =157 ($C_8H_{14}O_3$) ions were ketoacids, that did not possess a cyclobutyl (C_4) ring; DBE=2. Neutral losses of H_2O and CO_2 strongly indicated the presence of carboxylic moiety (pathways A and B).[6, 7] Neutral loss of water after decarboxylation can be explained by the elimination of H_2O from carbonyl moiety via a mechanism similar to the elimination of water from monomethyl esters of dicarboxylic acids.[6] As already discussed, a neutral loss of 58 Da, likely corresponding to a neutral loss of acetone via charge-remote McLafferty-like rearrangement [8, 9] indicates a presence of a “terminal” acetone moiety. The loss of 46 mass units corresponds to the charge-remote dissociation of the carboxylic group and is attributed to the neutral loss of formic acid. [6] Fragmentation

of the two isomeric products was nearly identical; hence, only one spectrum is shown in Fig. S4A. Given the above-discussed mechanisms, two possible isomers of these compounds are shown in Fig. S5.

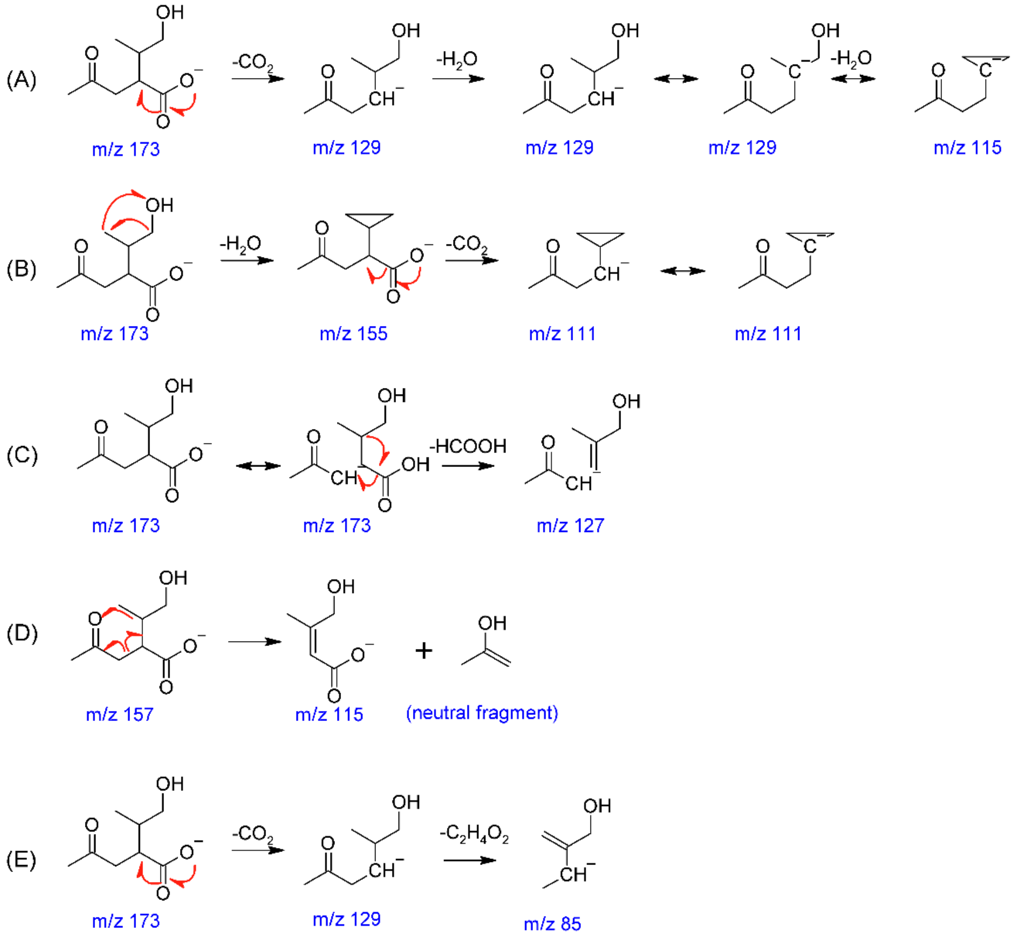

Figure S6 Proposed fragmentation mechanism compound with MW=174 Da

A single product detected as m/z 173 ($C_8H_{14}O_4$) ion was also tentatively identified as a ketoacid, due to characteristic neutral losses of H_2O, CO_2, and a neutral loss of acetone, following a McLafferty-like rearrangement. [8, 9] As in the case of the two ketoacids with MWs=158, a neutral loss of HCOOH was also observed. [8, 9] Formation of m/z 155 and 111 ions can be explained by decarboxylation (Fig. S6B) combined with a loss of water, which is accompanied by the formation of cyclopropane ring, as previously concluded.[11]

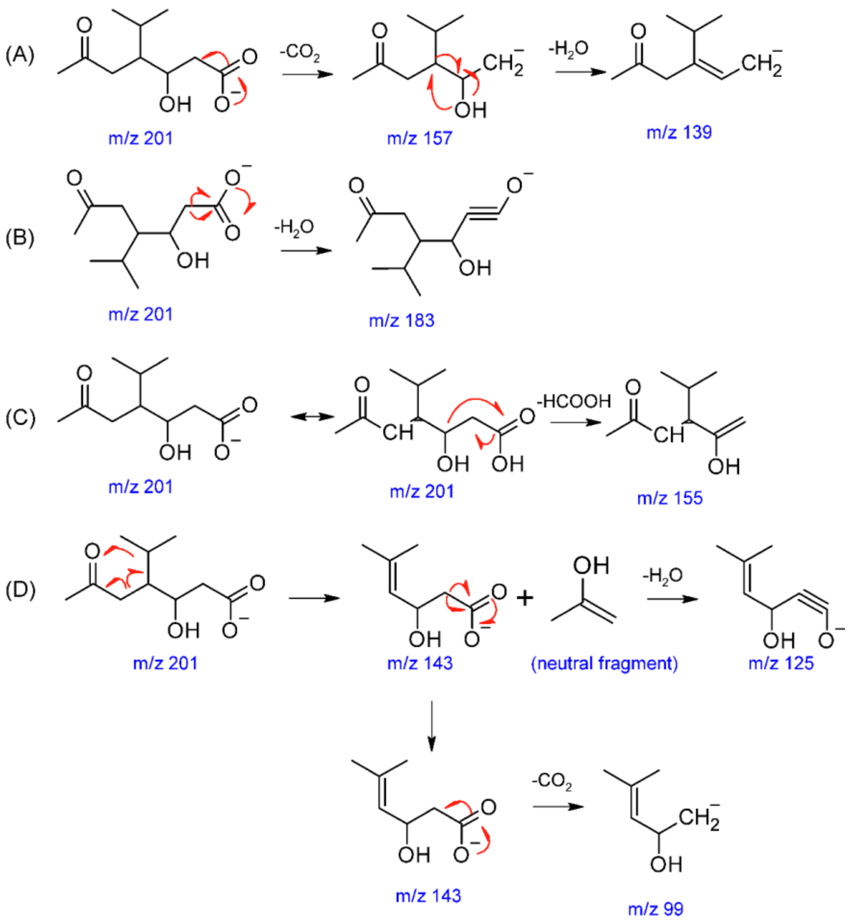

Figure S7 Proposed fragmentation mechanism compound with MW=202 Da

As in the case of the compound with MWs 158 and 174 Da, the formation of all observed ions can be explained by previously described mechanisms, characteristic of functionalized carboxylic acids. Based on the MS data acquired, it is impossible to unambiguously identify a specific isomer (especially the branching of the carbon side chain). However, taking into account that this product is likely formed following OH attack on the α-pinene cyclobutyl ring, [12-14] a tentative structure is proposed.

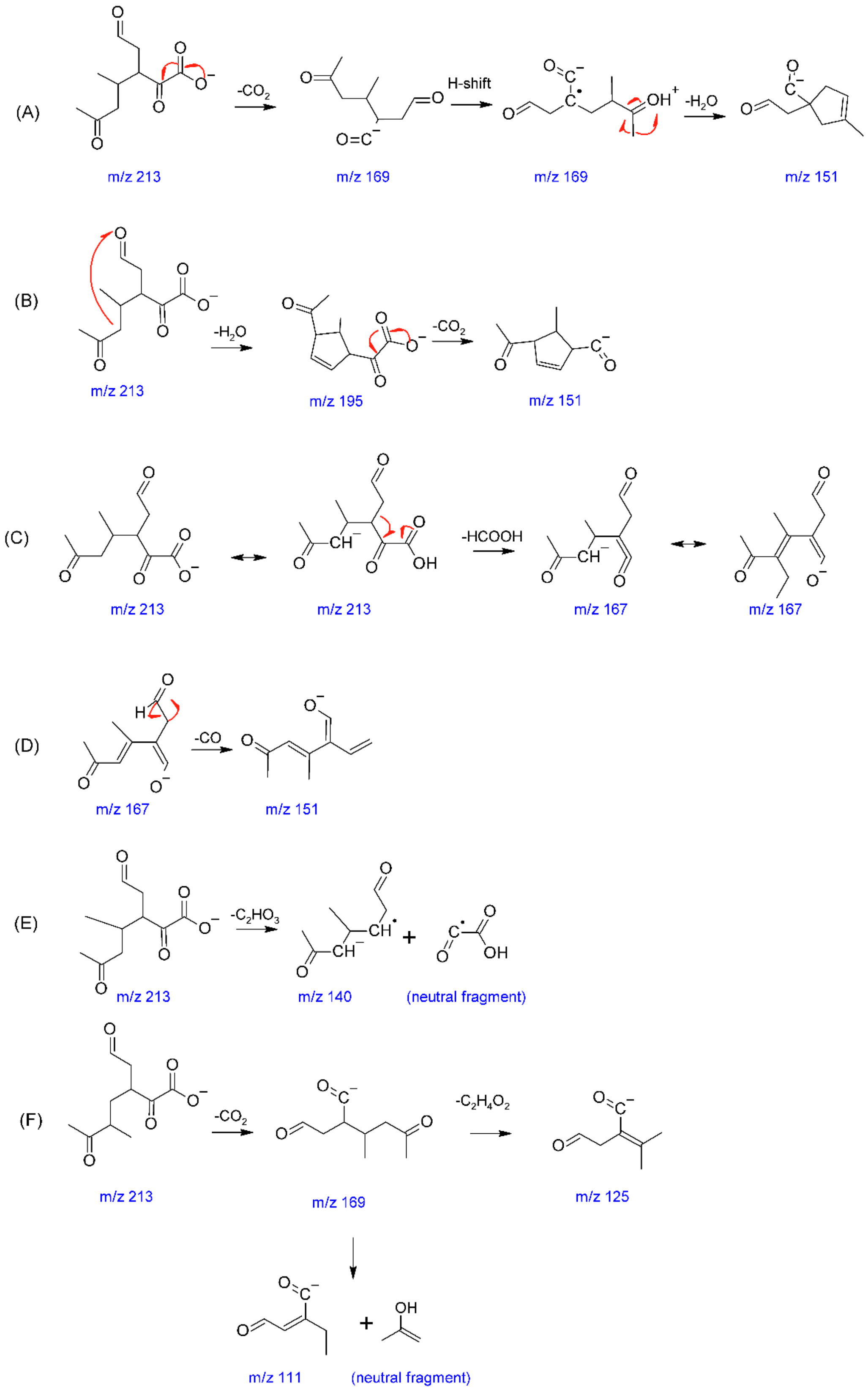

Figure S8 Proposed fragmentation mechanism compound with MW=214 Da

Two products detected as m/z 213 ($C_8H_{14}O_5$) ion were tentatively identified as dioxohydroxy carboxylic acids. Aside from the characteristic neutral losses of H_2O, and CO_2,[6] the fragmentation spectra of these two isomers show some unique features. Neutral loss of CO (28 Da) was attributed to

the presence of aldehyde moiety, which is consistent with the previously published data [9] as well as with the structures of the products with MWs 158 and 174 Da that were discussed earlier in this section. A second characteristic fragmentation is a neutral loss of 73 Da, which cannot be attributed to a peracid or a methyl ester. [6, 15] Based on the formation of this fragment, the product with MW=214 Da is proposed to be α-ketoacid (Fig. 6SE). Furthermore, the elimination of 88 Da (Fig. S4B) argues against the presence of two carboxylic groups (see also Table S3) and is tentatively attributed to the neutral loss of acetaldehyde (Fig S5F) combined with decarboxylation. Furthermore, this mechanism seems to be a unique feature of polycarbonyl acids, which would also explain why it was not observed for the products with MWs 157, 173, and 202 Da. Note however that the detailed data about negatively charged ions of functionalized carboxylic acids in ESI is extremely limited, [6, 10, 16] partially also due to the limited number of available standards. The possible fragmentation mechanisms shown in Fig. S8 can be also applied to the second possible isomer of this product (see Table 1 in the main text).

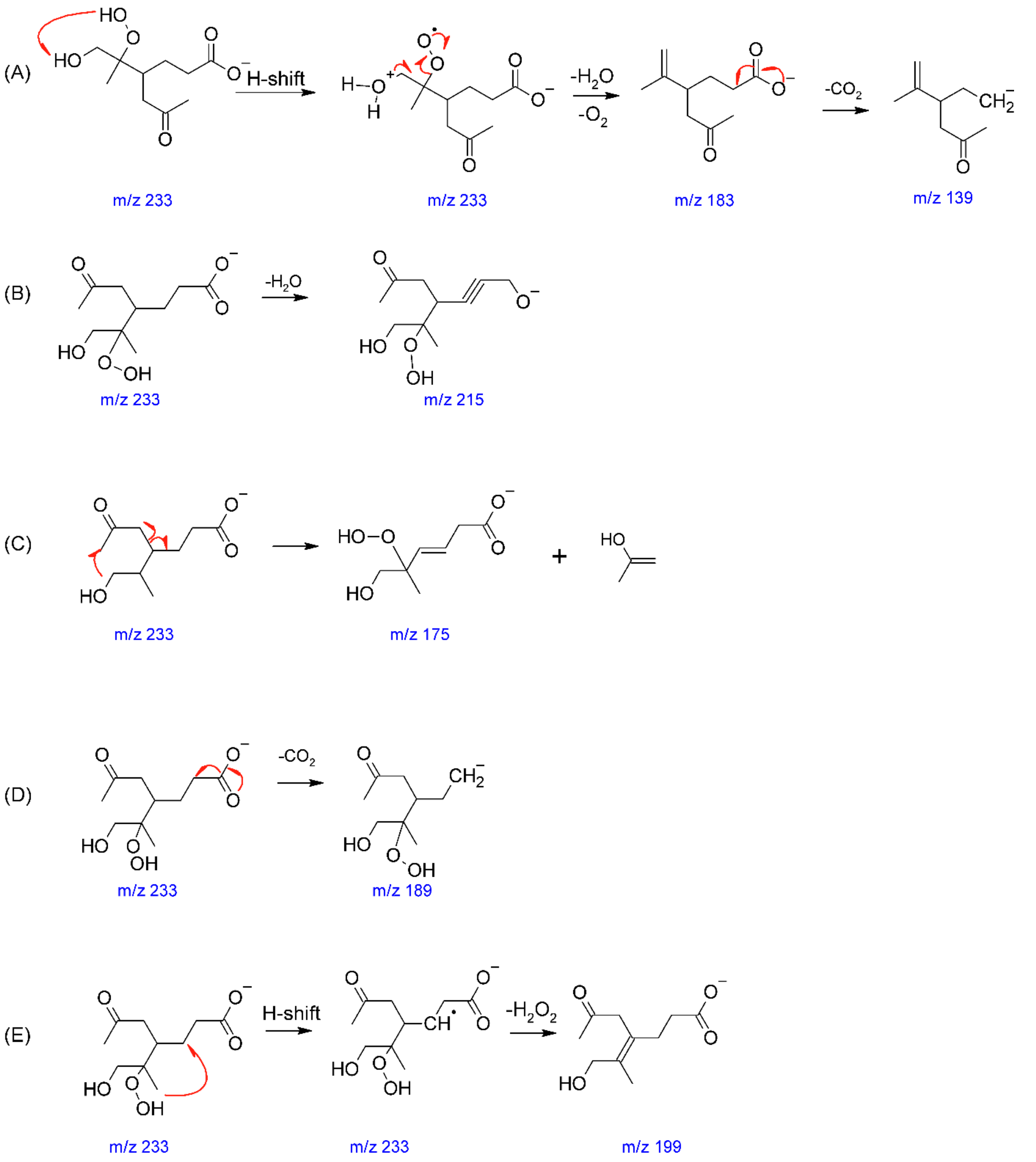

Figure S9 Proposed fragmentation mechanism compound with MW=233 Da

Aside from the neutral losses of H_2O and CO_2, attributed to the presence of carboxyl moiety, [6,7] fragmentation spectrum of the product detected as m/z 233 ($C_{10}H_{18}O_6$) ion exhibited some unique features, most notably neutral losses of 50 and 34 Da. A neutral loss of 34 Da is often ascribed to the hydroperoxyl moiety.[17] Compound with MW=234 Da was previously formed following aqueous oxidation of *cis*-pinonic acid by the OH and was tentatively identified as hydroperoxyl hemiacetal. However, the fragmentation spectrum shown in Fig. S4E was different as compared with the fragmentation spectrum acquired in our previous study of *cis*-pinonic acid oxidation by the OH.[12] Similar product was observed in our previous work from the gas-phase ozonolysis of limonene and was tentatively ascribed to a stable hydrate of the 2-hydroperoxy limononic acid.[17]

Based on the acquired MS data, the structure of the product detected as m/z 233 ion was proposed as a cyclobutyl-ring opening product containing hydroperoxy, hydroxy and carbonyl moieties.

The elimination of 34 Da may likely correspond to the elimination of H_2O_2 following a McLafferty-like rearrangement (Fig. S9E). Furthermore, the elimination of 50 Da can be explained by combined neutral losses of water and oxygen, which in turn may indicate the proximity of hydroxyl and hydroperoxyl moieties (Fig. S9E). Formation as a small but present fragment ion with m/z = 175 Da points indicated the presence of a “terminal” acetone moiety (Fig. S9C).

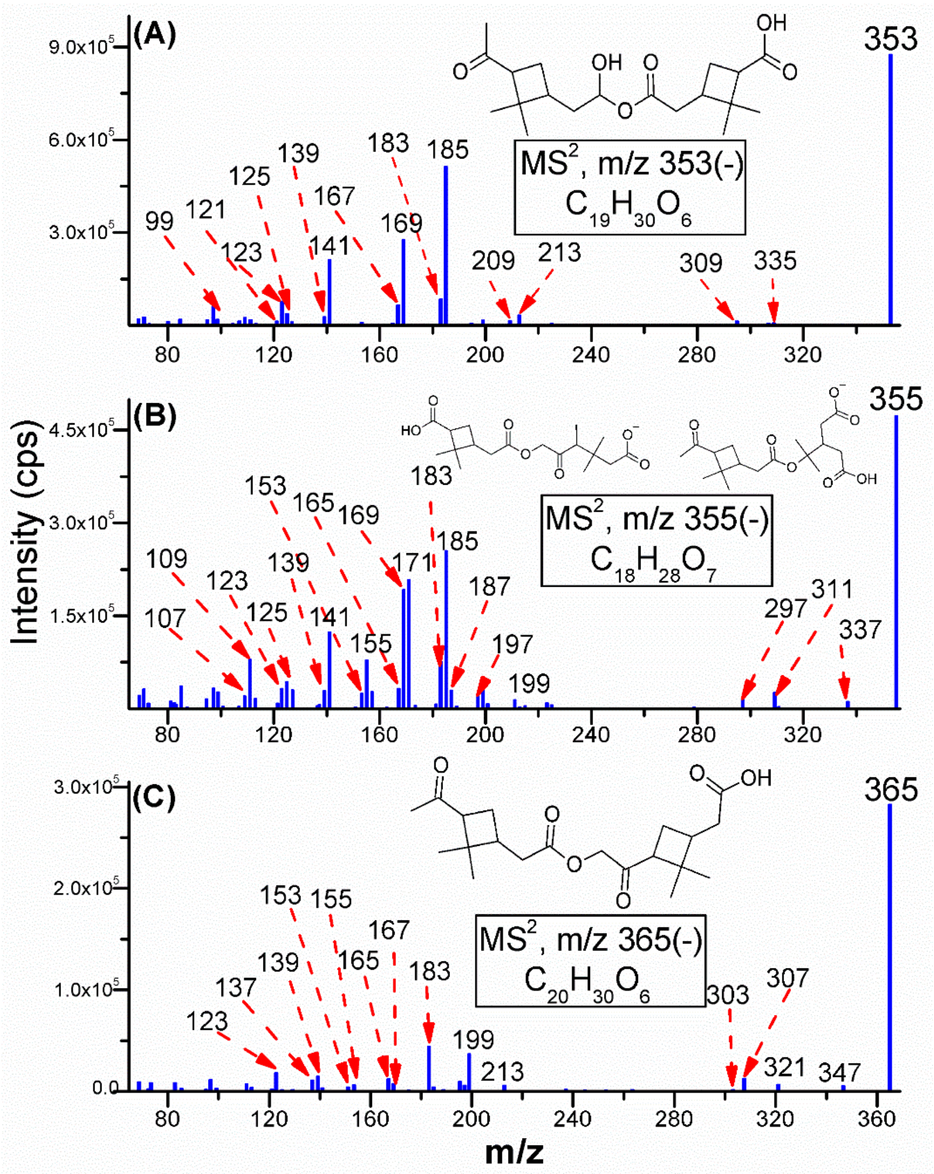

Figure S10 Fragmentation spectra for dimers with MW= 354 Da (A), 356 Da (B), and 365 Da (C)

Fragmentation spectra for dimers detected in monoterpenoic SOAs are primarily interpreted by proposing the dissociation of an ester bond combined with the regeneration of the $[M\text{-}H]^-$ for their “monomeric” building blocks. [17-21] This commonly accepted mechanism involves fragmentation of an ester bond via short or long-range McLafferty-like rearrangements. [10, 18]

The proposed fragmentation mechanism for the dimer with MW 354 tentatively identified as diol ester is presented in Fig. S11.

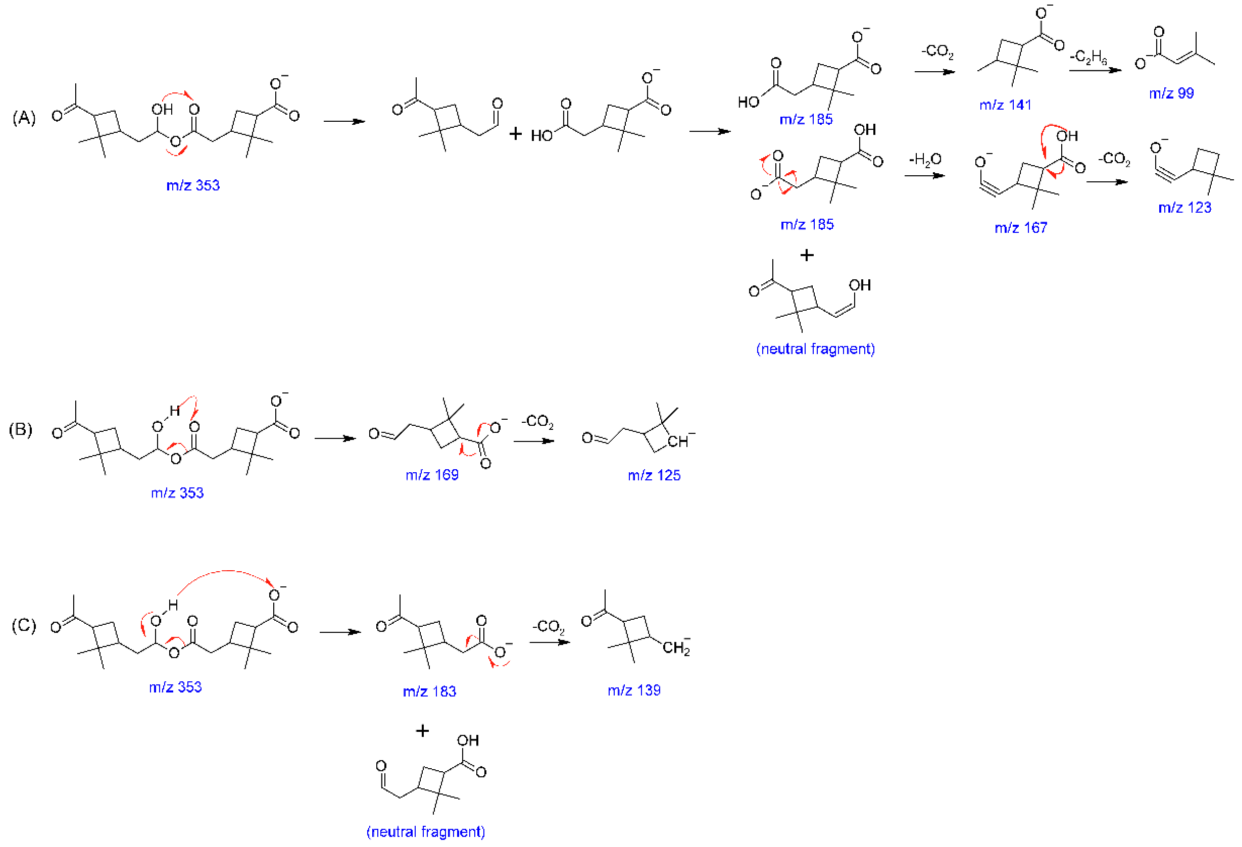

Figure S11 Proposed fragmentation mechanism for the dimer with MW 354 Da

Neutral losses of CO_2 and H_2O from the $[M\text{-}H]^-$ carboxylate anions of the dimer with MW=354 Da point out the presence of carboxylic moiety.[6, 7] As presented in Fig. S11, the m/z 169 and 185 fragment ions are formed following charge-remote dissociation of an ester bond.[10, 20] Because this dimer is proposed to be a hydrate-ester, [22 23] it allows a transfer of the negative charge across the ester bond following a long-range H atom shift (Fig. S11B),[10] without the presence of an electronegative atom (oxygen), the dissociation of the ester bond would likely result in the formation of a C=C bond.[11, 18, 19]

Formation of the fragment ions with lower m/z can be explained via neutral losses of CO_2 and H_2O from the “monomer” ions, formed via the fragmentation of an ester bond. [10]

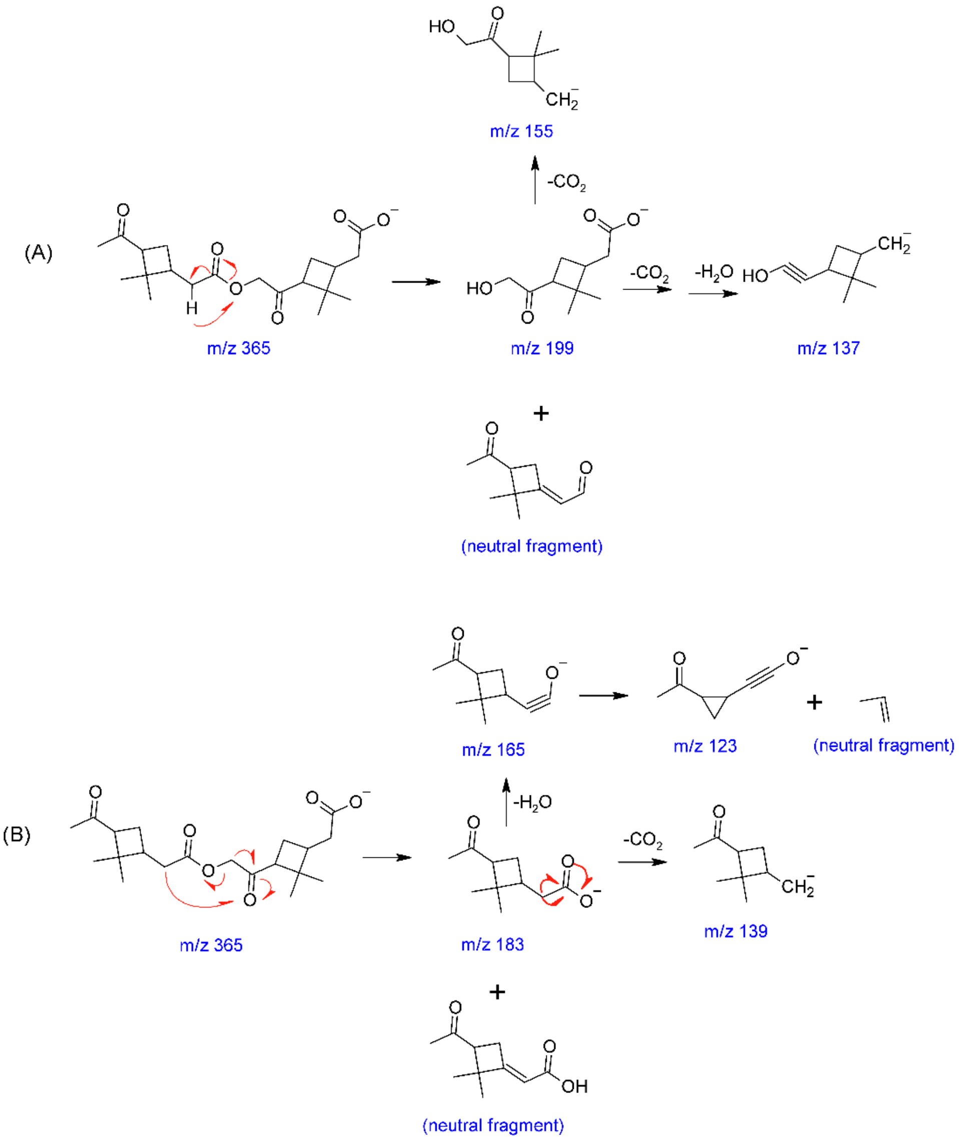

Figure S12 Proposed fragmentation mechanism for the dimer with MW 366Da

Neutral losses of CO_2 and H_2O from the $[M\text{-}H]^-$ were also observed for this dimer, thereby suggesting the presence of a carboxylic moiety. [6, 7] Fragmentation of an ester bond yields, [10, 18] yielded fragmentation ions with m/z 199 and 183.[11] The second-generation of the fragmentation ions are likely formed following the neutral losses of CO_2 and H_2O from these ions, yielding the fragments that were previously observed for 10-hydroxypinonic acid and *cis*-pinonic acid.[11, 24] Interestingly, elimination of 42 Da from the m/z 183 ion is a characteristic feature of the fragmentation spectrum of cis-pinonic acid, as previously discussed, [24, 25] thereby strongly indicating that it was one of the building blocks of the dimer with MW=366 Da.

The fragmentation spectrum shown in Fig. S10B was noticeably different as compared with the fragmentation spectra of the other two dimers, tentatively identified as acidic as the fragmentation spectra of the other two dimers. The structure of the dimer with MW=356 Da ($C_{18}H_{28}O_7$) was previously proposed as acidic α-acyloxyalkyl hydroperoxide, formed from the reaction of the C_9 SCI with pinic acid, [26] or a hydroperoxide dimer.[18] Formation of stable α-acyloxyalkyl hydroperoxides has been

previously proposed in a number of studies, investigating the composition of α-P_{SOA} with MS or hyphenated techniques (LC/MS).[27] However, currently available data strongly indicates that such molecules undergo rapid hydrolysis in the aqueous media, [23, 28] which argues against detecting α-acyloxyalkyl hydroperoxides in filter extracts via RP-LC/MS, that routinely uses aqueous eluents.

On the other hand, the structures of the hydroperoxide dimer, which was previously proposed for the dimer with MW=356 Da, [18] was not supported by the acquired experimental data. No convincing justification for the proposed structure was presented and fragmentation spectrum for this molecule also wasn't discussed.[18]

Due to large number of fragment ions, the formation of all of these fragments (Fig. 8B) cannot be explained by dissociation of an ester bond of a single dimer. Hence, it is proposed that the two isomeric dimer esters produced this complex fragmentation spectrum due to co-elution – Fig. S13. Some co-elution was observed for the majority of the dimers detected in this work because, under the experimental conditions used, obtained the baseline separation for all oligomers detected was not possible (Table S1). It is therefore reasonable to assume that isobaric dimers might be co-eluting under the LC/MS analysis conditions used.

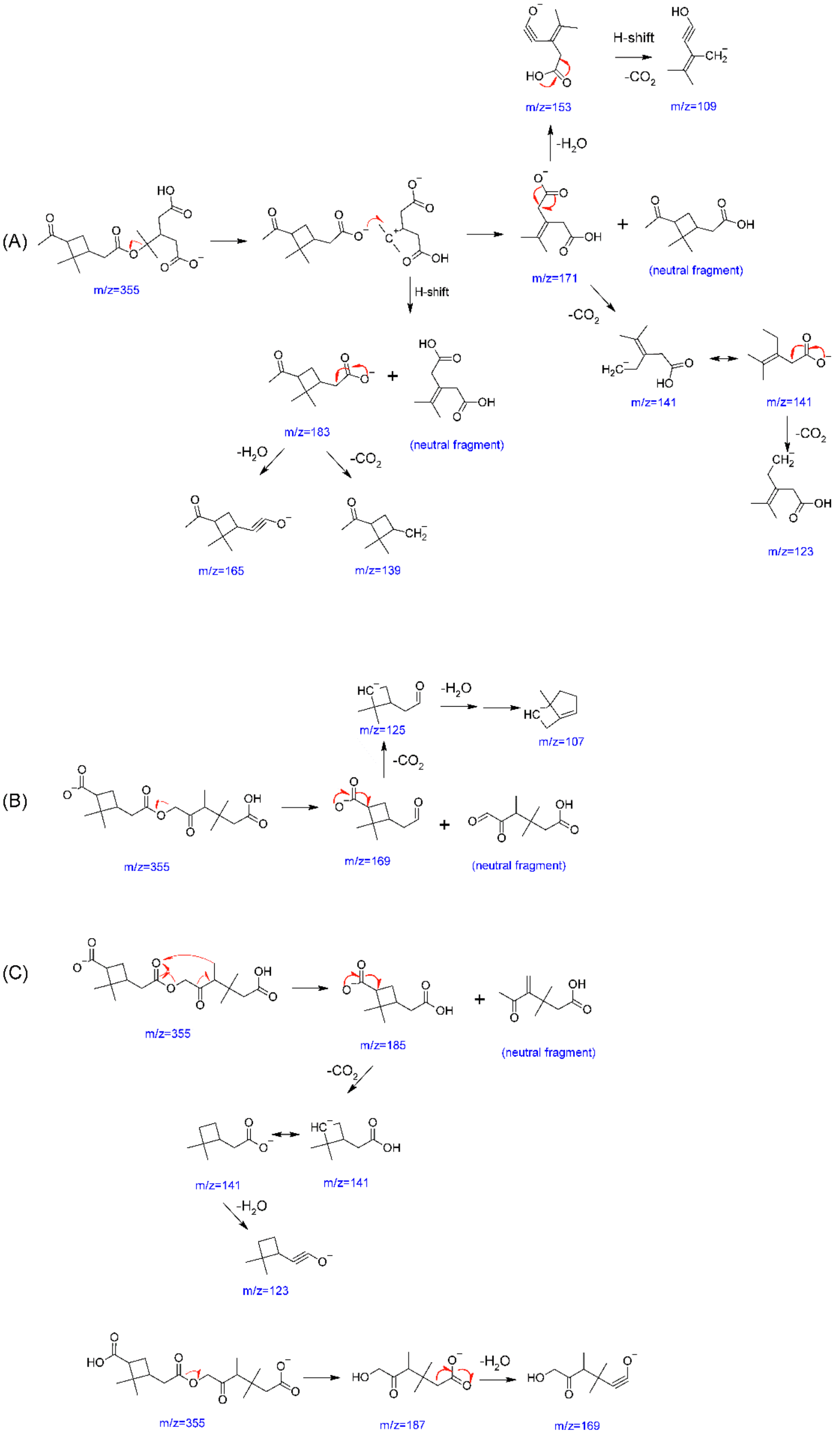

Figure S13 Proposed fragmentation mechanism for the two isomeric dimers with MWs = 356 Da

It is likely that both isomers of the dimer with MW=356 Da possessed carboxylic moiety, due to the relatively abundant fragment ions, formed following a characteristic neutral losses of CO_2 and H_2O from the $[M-H]^-$ pseudomolecular ion(s) [6, 7] - Fig. S13A. Diaterpenylic and *cis*-pinonic acid are proposed as the building blocks of the first isomeric dimer ester, which explains the presence of the characteristic m/z=171 and 183 fragment ions [19, 29]. As in the case of the acidic ester dimers with MWs, the formation of these main fragment ions can be explained by charge-remote dissociated of the ester bond [10]. Furthermore, the formation of lower-MW fragmentation ions can be rationalized via neutral losses of CO_2 and H_2O from these ions, following the above described mechanisms. Likewise for the second isomer, composed from cis-pinonic acid and a cyclobuty ring-opening acid with MW= 188 Da, the dissociated of an ester bond yields fragment ions with m/z =185 and 169. Likewise, the formation of the lower m/z fragment ions can again be rationalized via the elimination of CO_2 and H_2O from these ions.

S5. Estimation of k_{OH} values with empirical structure-activity relationship parameters

k_{OH} ($M^{-1}s^{-1}$) values for each molecule listed in Table 1 were estimated at 298 K using the empirical SAR parameters (Table S4), as previously described.[3, 30, 31] Based on the results presented in our previous study [12], hydration (formation of gem-diols) of the detected ketoacids was assumed to be negligible [32].

Table S4 Base rate coefficients and the values of neighboring parameters

**Group**	**Neighboring parameters**	
	**α-position**	**β- position**
CH_3	1.33	1.17
CH_2	1.14	1.08
CH	1.11	1.05
C	1.00	1.00
FC=O	0.22	0.90
COOH	0.16	0.59
COO-	0.54	0.64
OH	2.10	0.44
**Group**	**Base rate coefficient at 298 K ($M^{-1}s^{-1}$)**	
CH3	3.50E+08	
CH2	6.50E+08	
CH	4.70E+08	
OH	6.90E+07	

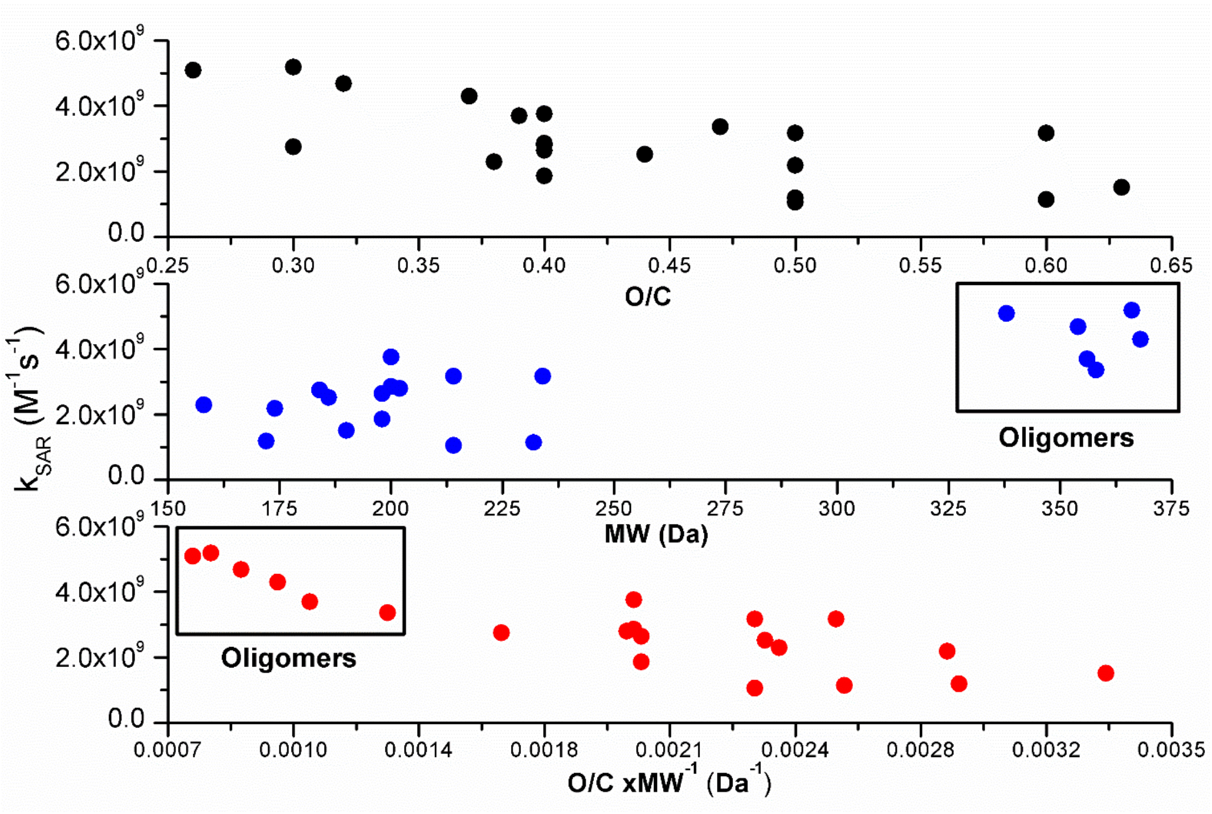

Figure S14 Estimated k_{SAR} values as a function of O/C ratios, MWs and O/C ratios normalized by MWs

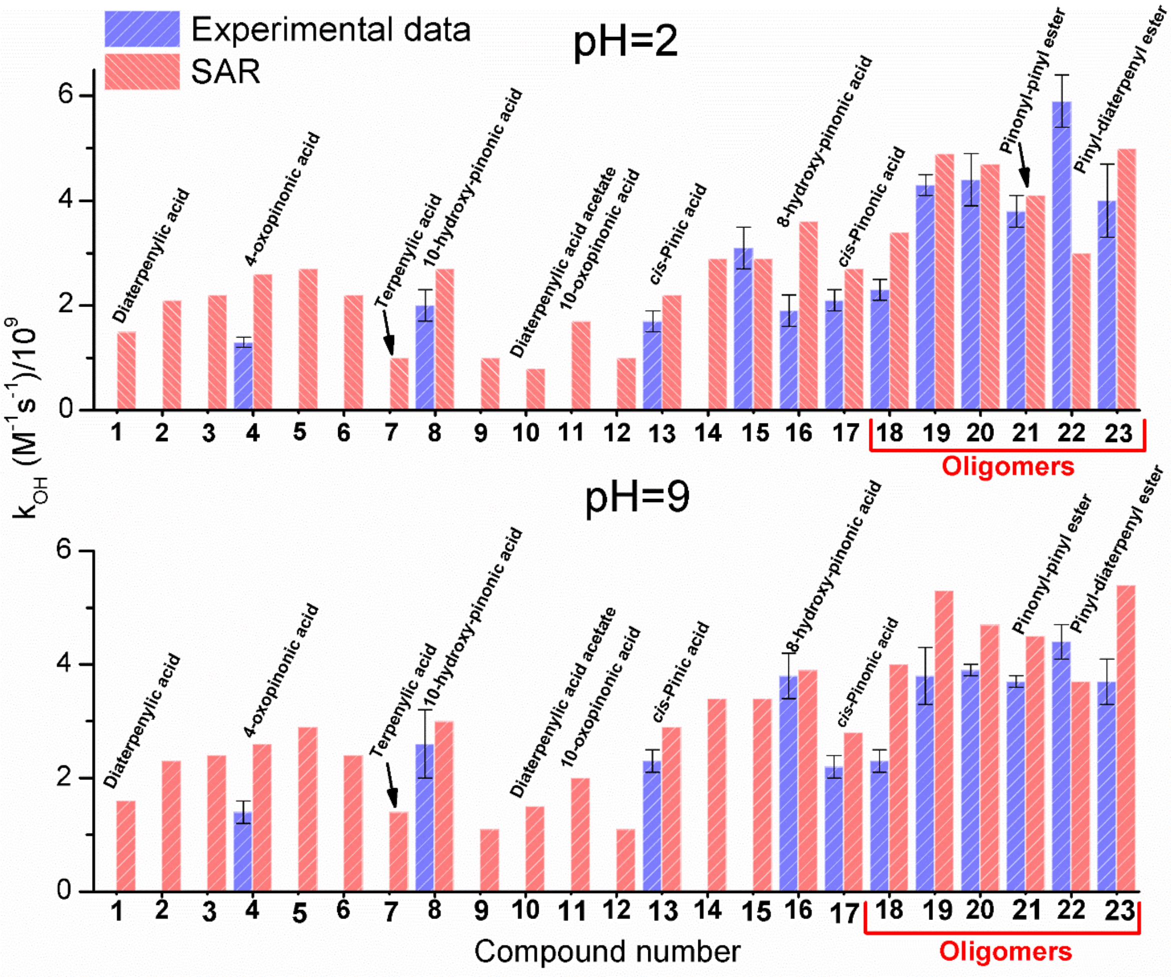

Figure S15 Measured and predicted k_{OH} values for the compounds listed in Table 1 in the main text. Absence of the measured values indicates formation of a given molecule during OH aging, resulting in an increase in the concentration.

S6. Kinetic box-model

Table S5 k_{OH} values used in the kinetic model

**No.**	**Name**	**Symbol**	**k_{OH} (M^{-1} s^{-1}) ×10^{-9}**	**Ref.**
1	Diaterpenylic acid	MW189	1.5	SAR
2	2-(1-hydroxypropan-2-yl)-4-oxopentanoate	MW173	2.2	
3	3-ethyl-5-oxohexanoic acid	MW157	2.3	
4	4-oxopinonic acid	MW197	2.6	
5	Hydroxy keto 4-isopropyl hexanoic acid	MW201	2.8	
6	4-oxo-2-(propan-2-yl)pentanoic acid	MW157_1	2.3	
7	Terpenylic acid	MW171	1.2	
8	10-Hydroxy-pinonic acid	MW199	2.9	
9	4-methyl-2,6-dioxo-3-(2-oxoethyl)heptanoic acid	MW213	1.1	
10	Diaterpenylic acid acetate	MW231	1.2	
11	10-oxopinonic acid	MW197_1	1.9	
12	2,6-dioxo-3-(1-oxopropan-2-yl)heptanoic acid	MW213_1	1.1	
13	*cis*-Pinic acid	MW185	2.5	
14	4,4-dimethyl-trioxoheptanoic acid	MW213	3.2	
15	4,4,5-trimethyl-hydroxy-hydroperoxyl-oxoheptanoic acid	MW233	3.2	
16	8-Hydroxy-pinonic acid	MW199_1	3.8	
17	*cis*-Pinonic acid	MW183	2.8	

Table S5 Kinetic data used in the box-model, continued…

**No.**	**Name**	**Symbol**	**k_{OH} (M^{-1} s^{-1}) ×10^{-9}**	**Ref.**

18	Aldol reaction product, *cis*-pinonic acid and norpinonaldehyde	MW355	3.7	Average measured values (this work)
19	Pinic acid and hydrated pinonaldehyde ester	MW337	4.1	
20	Pinonyl-pinyl ester	MW353	4.2	
21	Pinyl-diaterpenyl ester	MW367	3.8	
22	Hydroxy-pinonic acid-pinonic ester	MW357	5.2	
23	Aldol reaction product, *cis*-pinonic acid and norpinonaldehyde	MW365	3.9±0.5	

Table S6 Reactions included in the kinetic box-model

**Reaction number**	**Reaction**[a]	**k (M^{-1} s^{-1})**
1	H2O2 = OH + OH	$1\times\times10^{-5}$
2	OH + H2O2 = HO2	2.7×10^{7}
3	HO2+H2O2 = OH	3.7
4	HO2 + HO2 = H2O2	8.3×10^{5}
5	OH + HO2 = H2O+O2	7.1×10^{9}
6	MW189 + OH= prdc	1.52×10^{9}
7	MW173 + OH= prdc	2.19×10^{9}
8	MW157 + OH= prdc	2.29×10^{9}
9	MW197 + OH= prdc	2.64×10^{9}
10	MW201 + OH= prdc	2.80×10^{9}
11	MW157_1 + OH= prdc	2.29×10^{9}
12	MW171 + OH = prdc	1.19×10^{9}
13	MW199 + OH = prdc	2.86×10^{9}
14	MW213 + OH = prdc	1.05×10^{9}
15	MW231 + OH = prdc	1.15×10^{9}
16	MW197_1 + OH = prdc	1.86×10^{9}
17	MW213_1 + OH = prdc	1.05×10^{9}
18	MW185 + OH = prdc	1.29×10^{9}
19	MW185 + OH = MW189	1.2×10^{8}
20	MW185 + OH = MW157	2×10^{7}
21	MW185 + OH = MW157_1	5×10^{7}
22	MW185 + OH = MW171	8.9×10^{8}
23	MW185 + OH = MW231	3×10^{8}
24	MW213 + OH = prdc	3.17×10^{9}
25	MW233 + OH = prdc	3.17×10^{9}
26	MW199_1 + OH = prdc	3.24×10^{9}
27	MW199_1 + OH = MW173	2.1×10^{8}
28	MW199_1 + OH = MW213+ MW213_1	3.5×10^{8}

29	MW183 + OH = prdc	1.55×10^{9}
30	MW183 + OH = MW199+ MW199_1	4.5×10^{8}
31	MW183 + OH = MW197_1	4.5×10^{8}

Table S6 Reactions included in the kinetic box-model, continued…

32	MW183 + OH = MW231	3×10^{8}
33	MW355 + OH = prdc	2.3×10^{9}
34	MW337 + OH = prdc	4.1×10^{9}
35	MW353 + OH = prdc	4.2×10^{9}
36	MW367 + OH = prdc	3.8×10^{9}
37	MW357 + OH = prdc	5.2×10^{9}
38	MW365 + OH = prdc	3.9×10^{9}
39	MW168 + OH= MW183	1.25×10^{9}
40	MW168 + OH = prdc	1.25×10^{9}
41	MW154 + OH= MW185	1.7×10^{9}
42	MW154 + OH = prdc,	3×10^{8}

In Table S6, reactions 1-5 describe generation of the steady-state concentration of OH via photolysis of H_2O_2. [33] The rate coefficients of reaction 1 in Table S6 is an empirical values, obtained by fitting the observed photolysis rate of H_2O_2, in the absence of organic reactants. The temporal concentration of H_2O_2 during photolysis was obtained by titrating the aliquots of the reacting solution with potassium permanganate.

Several reactions are listed in Table S6 for precursors (see Table 2 in the main text), which reflects their conversion in the detected OH aging markers with different yields.

Table S7 Initial concentrations of the reactants used in the kinetic box-model

**Name**	**Initial concentration (M)**	**Name**	**Initial concentration (M**
H_2O_2	0.011	MW185	1.4×10^{-4}
MW189	1.8×10^{-6}	MW213	2.2×10^{-6}
MW173	3.0×10^{-6}	MW233	2.4×10^{-6}
MW157	1.9×10^{-6}	MW199_1	1.5×10^{-4}
MW197	3.2×10^{-6}	MW183	2.0×10^{-5}
MW201	6.5×10^{-7}	MW355	1.7×10^{-6}
MW157_1	4.1×10^{-7}	MW337	2.1×10^{-5}
MW171	1.3×10^{-5}	MW353	1.6×10^{-5}
MW199	2.4×10^{-5}	MW367	1.8×10^{-5}
MW213	1.4×10^{-6}	MW357	5.3×10^{-7}
MW231	7.5×10^{-6}	MW365	3.3×10^{-6}
MW197_1	8.1×10^{-6}	MW168	2.0×10^{-5}
MW213_1	2.2×10^{-6}	MW154	2.0×10^{-5}

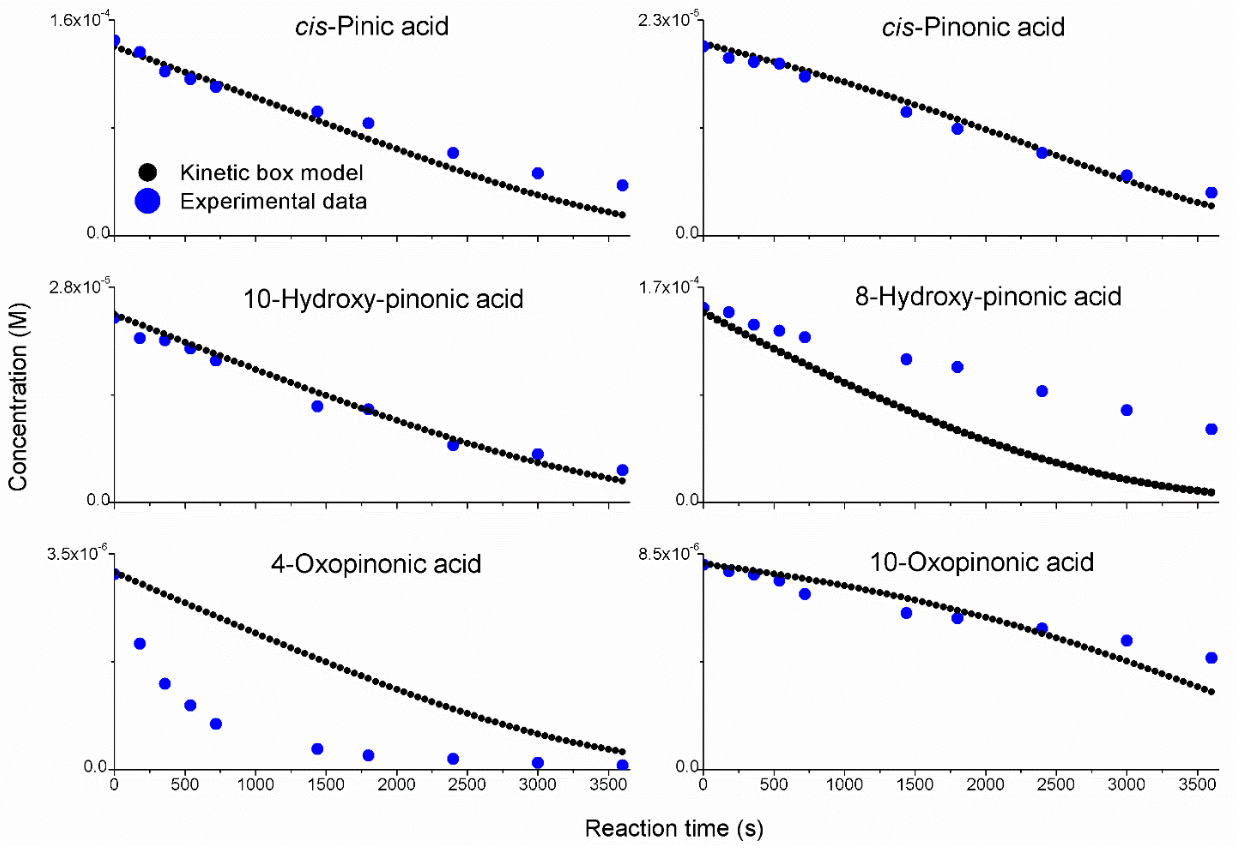

Figure S16 Experimental and modeled temporal concentration profiles of the early-stage products of ozonolysis of α-pinene during aqueous aging by the OH

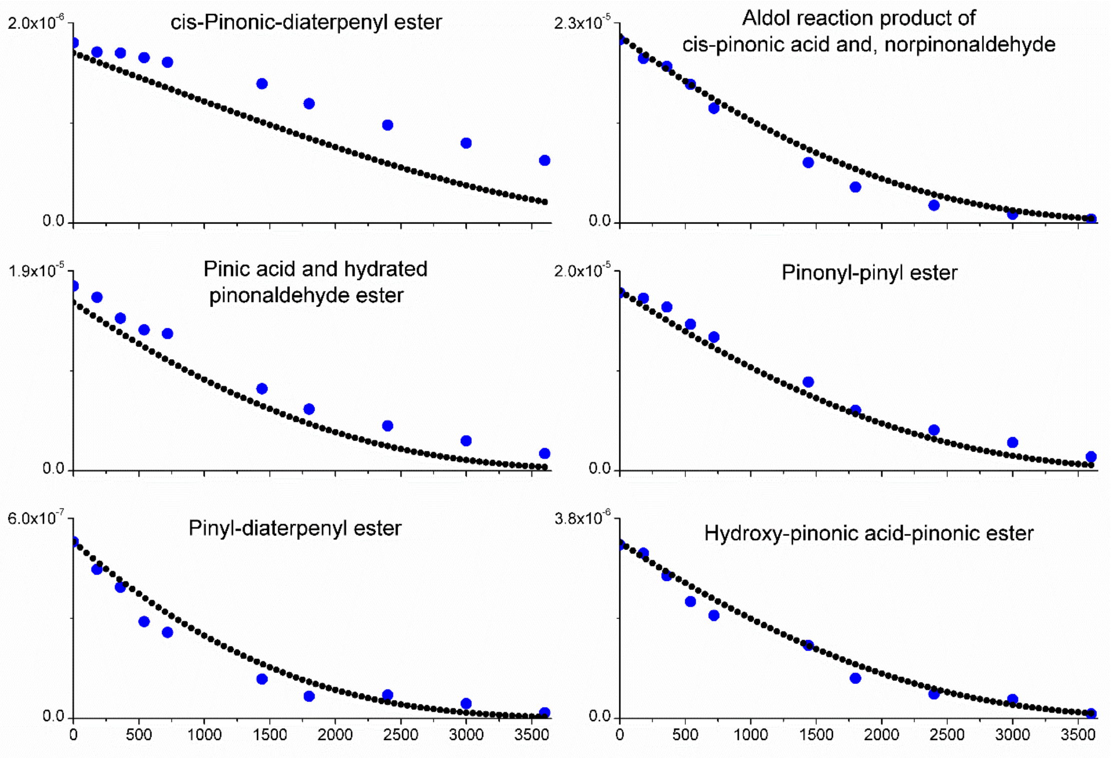

338

339 **Figure S17** Experimental and modelled temporal concentration profiles of the oligomers formed by the gas-phase
340 ozonolysis of α-pinene during aqueous oxidation by the OH

341

342

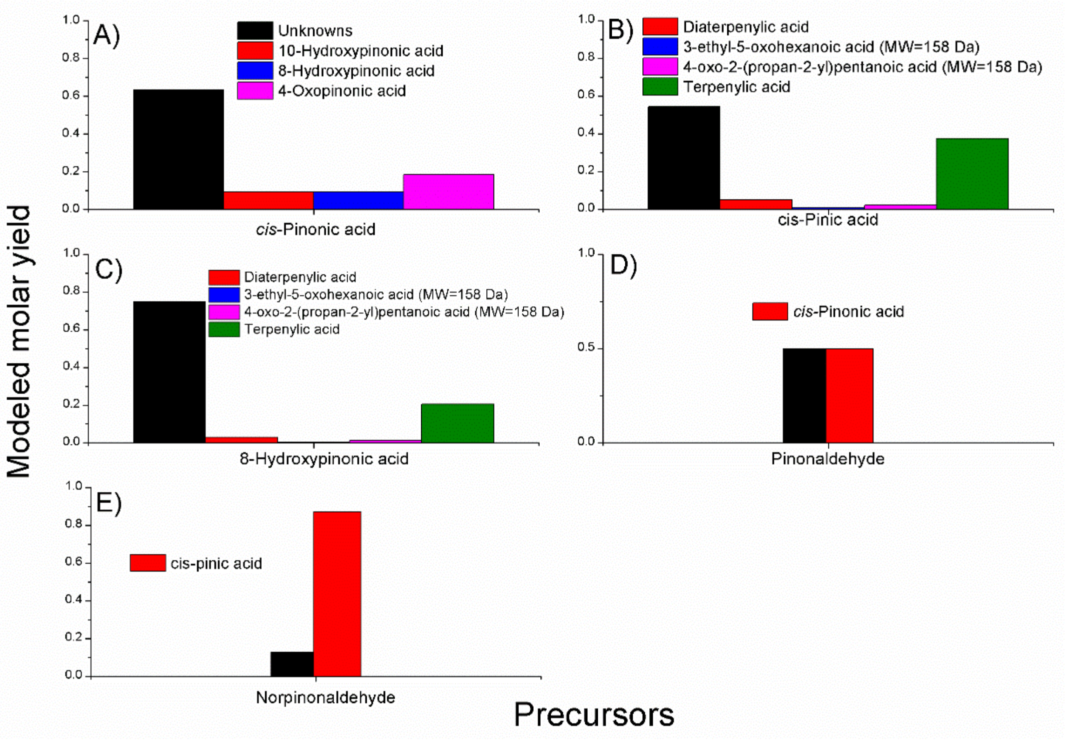

Figure S18 Modeled yields of the terpenoic acid under investigation from the major components of α-pinene SOA

Table S8 Bimolecular reaction rate coefficients for the reaction of terpenoic precursors under investigation with the OH in the gas and aqueous phase and their dimensionless Henry's law constant values

**Name**	$k_{OH_{aq}}$ **($M^{-1}s^{-1}$) ×10^{-9}**	k_{OH_g} **($M^{-1}s^{-1}$) ×10^{-9}**	**H^{cc} ×10^{-9}**	k_{OH_g} **($cm^3 molec^{-1}s^{-1}$) ×10^{12}**
Early-stage, lower-MW products				
cis-Pinic acid	2.55	2.52	4.68	4.23
cis-Pinonic acid	2.30	2.75	0.05	3.83
10-Hydroxy-pinonic acid	4.33	2.86	0.35	7.18
8-Hydroxy-pinonic acid	4.73	3.76	0.35	7.85
4-oxopinonic acid	2.17	2.64	9.13	3.60
10-oxopinonic acid	11.78	1.86	5.03	19.56
Oligomers				
Two isomeric dimer esters; Cis-pinonic and diaterpenylic acids Pinic acid and hydroxy-keto acid (MW=188 Da)	5.92	2.30	7.49×10^5	9.83
Aldol reaction product, *cis*-pinonic acid and norpinonaldehyde	4.47	4.10	6.90×10^5	7.42
Pinic acid and hydrated pinonaldehyde ester	4.92	4.20	9.83×10^4	8.17
Pinonyl-pinyl ester	5.02	3.80	1.81×10^5	8.33
Pinyl-diaterpenyl ester	4.09	5.20	6.78×10^7	6.78
Hydroxy-pinonic acid-pinonic ester	4.26	3.90	2.00×10^3	7.08
Cyclobutyl-ring opening products				
Diaterpenylic acid	1.52	4.13	0.75	6.9
2-(1-hydroxypropan-2-yl)-4-oxopentanoate	2.19	2.00	0.14	3.3
3-ethyl-5-oxohexanoic acid	2.29	2.41	0.03	4.0
Hydroxy keto 4-isopropyl hexanoic acid	2.80	9.55	0.14	15.9

Table S8, continued…

**Name**	$k_{OH_{aq}}$ **(M^{-1}s^{-1}) ×10^{-9}**	k_{OH_g} **(M^{-1}s^{-1}) ×10^{-9}**	**H^{cc} ×10^{-9}**	k_{OH_g} **(cm^{3}molec^{-1}s^{-1}) ×10^{12}**
4-oxo-2-(propan-2-yl)pentanoic acid	2.29	2.41	0.034	4.0
Terpenylic acid	1.19	1.72	0.026	2.9
2,6-dioxo-3-(1-oxopropan-2-yl)heptanoic acid	1.05	11.9	4.57×10^4	19.7
Diaterpenylic acid acetate	1.15	4.08	1.20×10^3	6.8
4,4-dimethyl-trioxoheptanoic acid	1.05	11.9	4.57×10^4	19.7
4,4,5-trimethyl-hydroxy-hydroperoxyl-oxoheptanoic acid	3.17	11.9	4.57×10^4	19.7

Table S9 The data used to prepare a simplified kinetic model of the aqueous OH aging of α-P SOA

**Group of compounds**	**Measured k_{OH} (M^{-1} s^{-1}) ×10^{-9} at 298K**			**Relative concertation in fresh SOA**
	Average	Upper limit	Lower limit	
Ring-retaining	2.3	3.4	1.1	80%
Dimers	3.9	3	4.8	11%
Ring-opening[a]	2.5	4.2	0.7	9%

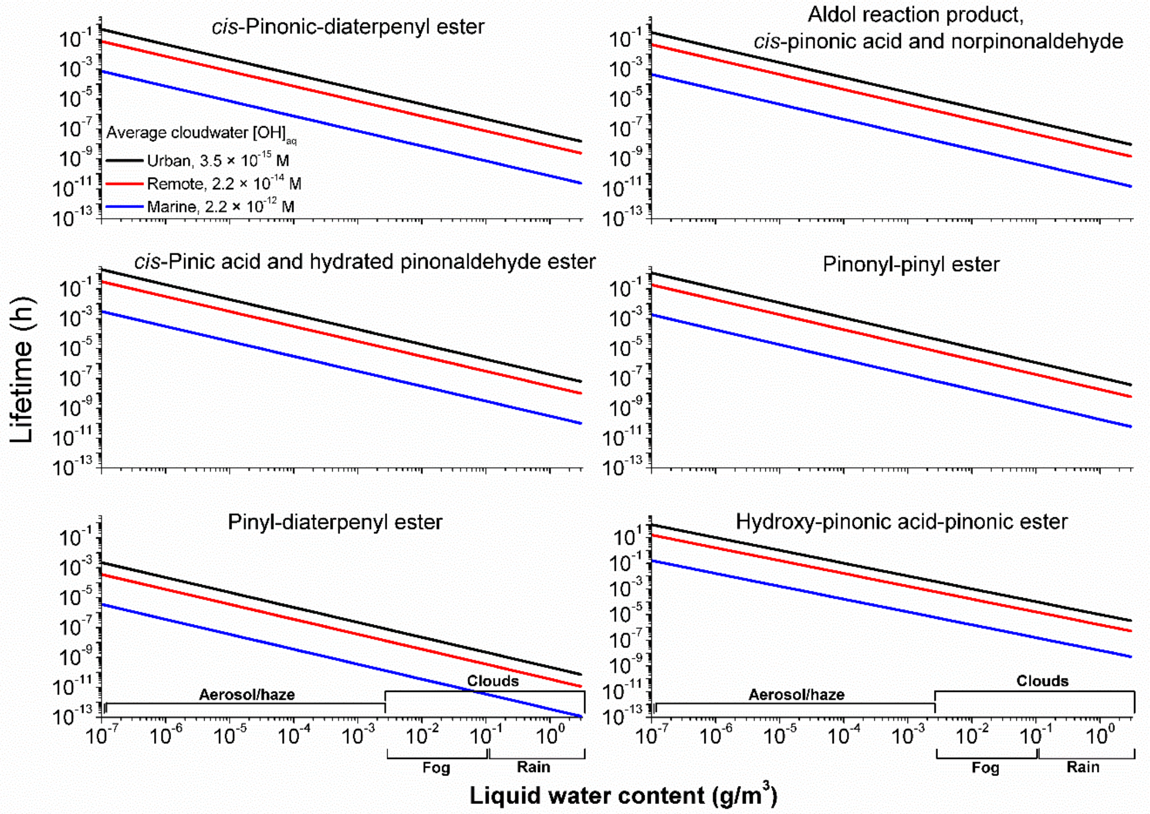

Figure S19 The estimated total lifetimes of the dimers due to the reaction with the OH in the gas and aqueous phases. The shorter, combined lifetime, decreasing with the increasing values of LWC shows that a given precursor undergoes aqueous processing under realistic atmospheric conditions. Colors represent average $[OH]_{aq}$ in different clouds.

Table S10 Measured and predicted pK_a values for the terpenoic acids investigated in this work

<table>
<tr><th>Compound number</th><th>O/C</th><th>MW</th><th>First pK_a</th><th>Ref.</th></tr>
<tr><td>1</td><td>0.63</td><td>190</td><td>3.95</td><td rowspan="9">[34]</td></tr>
<tr><td>2</td><td>0.5</td><td>174</td><td>3.95</td></tr>
<tr><td>3</td><td>0.38</td><td>158</td><td>4.68</td></tr>
<tr><td>4</td><td>0.4</td><td>198</td><td>3.34</td></tr>
<tr><td>5</td><td>0.4</td><td>202</td><td>4.58</td></tr>
<tr><td>6</td><td>0.38</td><td>158</td><td>4.63</td></tr>
<tr><td>7</td><td>0.5</td><td>172</td><td>4.25</td></tr>
<tr><td>8</td><td>0.4</td><td>200</td><td>4.44</td></tr>
<tr><td>9</td><td>0.5</td><td>214</td><td>3.21</td></tr>
<tr><td>10</td><td>0.6</td><td>232</td><td>3.93</td><td>[29]</td></tr>
<tr><td>11</td><td>0.4</td><td>198</td><td>4.3</td><td rowspan="2">[34]</td></tr>
<tr><td>12</td><td>0.5</td><td>214</td><td>3.21</td></tr>
<tr><td>13</td><td>0.44</td><td>186</td><td>4.48</td><td>[35, 36]</td></tr>
<tr><td>14</td><td>0.5</td><td>214</td><td>3.21</td><td rowspan="3">[34]</td></tr>
<tr><td>15</td><td>0.6</td><td>234</td><td>4.29</td></tr>
<tr><td>16</td><td>0.4</td><td>200</td><td>4.44</td></tr>
<tr><td>17</td><td>0.3</td><td>184</td><td>4.72</td><td>[29, 36]</td></tr>
<tr><td>18</td><td>0.39</td><td>356</td><td>3.8</td><td rowspan="6">[34]</td></tr>
<tr><td>19</td><td>0.26</td><td>338</td><td>4.3</td></tr>
<tr><td>20</td><td>0.32</td><td>354</td><td>4.17</td></tr>
<tr><td>21</td><td>0.37</td><td>368</td><td>3.7</td></tr>
<tr><td>22</td><td>0.47</td><td>358</td><td>4.3</td></tr>
<tr><td>23</td><td>0.3</td><td>366</td><td>4.11</td></tr>
</table>

References

1. Schaefer, T.; Wen, L.; Estelmann, A.; Maak, J.; Herrmann, H., pH- and Temperature-Dependent Kinetics of the Oxidation Reactions of OH with Succinic and Pimelic Acid in Aqueous Solution. **2020,** *11*, (4), 320.
2. Schöne, L.; Schindelka, J.; Szeremeta, E.; Schaefer, T.; Hoffmann, D.; Rudzinski, K. J.; Szmigielski, R.; Herrmann, H., Atmospheric aqueous phase radical chemistry of the isoprene oxidation products methacrolein, methyl vinyl ketone, methacrylic acid and acrylic acid – kinetics and product studies. **2014,** *16*, (13), 6257-6272.
3. Witkowski, B.; Chi, J.; Jain, P.; Błaziak, K.; Gierczak, T., Aqueous OH kinetics of saturated C6–C10 dicarboxylic acids under acidic and basic conditions between 283 and 318 K; new structure-activity relationship parameters. **2021,** *267*, 118761.
4. Joback, K. G.; Reid, R. C., Estimation of pure-component properties from group-contributions. **1987,** *57*, (1-6), 233-243.
5. Wilke, C. R.; Chang, P., Correlation of diffusion coefficients in dilute solutions. **1955,** *1*, (2), 264-270.
6. Grossert, J. S.; Fancy, P. D.; White, R. L., Fragmentation pathways of negative ions produced by electrospray ionization of acyclic dicarboxylic acids and derivatives. **2005,** *83*, (11), 1878-1890.
7. Bandu, M. L.; Watkins, K. R.; Bretthauer, M. L.; Moore, C. A.; Desaire, H., Prediction of MS/MS Data. 1. A Focus on Pharmaceuticals Containing Carboxylic Acids. **2004,** *76*, (6), 1746-1753.
8. Yasmeen, F.; Szmigielski, R.; Vermeylen, R.; Gómez-González, Y.; Surratt, J. D.; Chan, A. W. H.; Seinfeld, J. H.; Maenhaut, W.; Claeys, M., Mass spectrometric characterization of isomeric terpenoic acids from the oxidation of α-pinene, β-pinene, d-limonene, and Δ3-carene in fine forest aerosol. **2011,** *46*, (4), 425-442.
9. Organic Mass Spectrometry. In *Fundamentals of Contemporary Mass Spectrometry;* 2007; pp 195-261.
10. Demarque, D. P.; Crotti, A. E. M.; Vessecchi, R.; Lopes, J. L. C.; Lopes, N. P., Fragmentation reactions using electrospray ionization mass spectrometry: an important tool for the structural elucidation and characterization of synthetic and natural products. **2016,** *33*, (3), 432-455.
11. Yasmeen, F.; Vermeylen, R.; Maurin, N.; Perraudin, E.; Doussin, J.-F.; Claeys, M., Characterisation of tracers for aging of α-pinene secondary organic aerosol using liquid chromatography/negative ion electrospray ionisation mass spectrometry. **2012,** *9*, (3), 236-246.
12. Witkowski, B.; Gierczak, T., cis-Pinonic Acid Oxidation by Hydroxyl Radicals in the Aqueous Phase under Acidic and Basic Conditions: Kinetics and Mechanism. **2017,** *51*, (17), 9765-9773.
13. Enami, S.; Sakamoto, Y., OH-Radical Oxidation of Surface-Active cis-Pinonic Acid at the Air–Water Interface. **2016,** *120*, (20), 3578-3587.
14. Amorim, J. V.; Guo, X.; Gautam, T.; Fang, R.; Fotang, C.; Williams, F. J.; Zhao, R., Photo-oxidation of pinic acid in the aqueous phase: a mechanistic investigation under acidic and basic pH conditions. **2021,** *1*, (5), 276-287.
15. Steimer, S. S.; Kourtchev, I.; Kalberer, M., Mass Spectrometry Characterization of Peroxycarboxylic Acids as Proxies for Reactive Oxygen Species and Highly Oxygenated Molecules in Atmospheric Aerosols. **2017,** *89*, (5), 2873-2879.
16. Davoli, E.; Gross, M. L., Charge remote fragmentation of fatty acids cationized with alkaline earth metal ions. **1990,** *1*, (4), 320-324.
17. Witkowski, B.; Gierczak, T., Characterization of the limonene oxidation products with liquid chromatography coupled to the tandem mass spectrometry. **2017,** *154*, 297-307.
18. Zhang, X.; McVay, R. C.; Huang, D. D.; Dalleska, N. F.; Aumont, B.; Flagan, R. C.; Seinfeld, J. H., Formation and evolution of molecular products in α-pinene secondary organic aerosol. **2015,** *112*, (46), 14168-14173.

19. Yasmeen, F.; Vermeylen, R.; Szmigielski, R.; Iinuma, Y.; Böge, O.; Herrmann, H.; Maenhaut, W.; Claeys, M., Terpenylic acid and related compounds: precursors for dimers in secondary organic aerosol from the ozonolysis of α- and β-pinene. **2010,** *10*, (19), 9383-9392.

20. Muller, L.; Reinnig, M. C.; Warnke, J.; Hoffmann, T., Unambiguous identification of esters as oligomers in secondary organic aerosol formed from cyclohexene and cyclohexene/alpha-pinene ozonolysis. **2008,** *8*, (5), 1423-1433.

21. Gao, Y.; Hall, W. A.; Johnston, M. V., Molecular Composition of Monoterpene Secondary Organic Aerosol at Low Mass Loading. **2010,** *44*, (20), 7897-7902.

22. Iinuma, Y.; Boge, O.; Gnauk, T.; Herrmann, H., Aerosol-chamber study of the alpha-pinene/O-3 reaction: influence of particle acidity on aerosol yields and products. **2004,** *38*, (5), 761-773.

23. Witkowski, B.; Gierczak, T., Early stage composition of SOA produced by α-pinene/ozone reaction: α-Acyloxyhydroperoxy aldehydes and acidic dimers. **2014,** *95*, 59-70.

24. Witkowski, B.; Jurdana, S.; Gierczak, T., Limononic Acid Oxidation by Hydroxyl Radicals and Ozone in the Aqueous Phase. **2018,** *52*, (6), 3402-3411.

25. Witkowski, B.; Al-sharafi, M.; Gierczak, T., Kinetics and products of the aqueous-phase oxidation of β-caryophyllonic acid by hydroxyl radicals. **2019,** *213*, 231-238.

26. Hall, W. I. V.; Johnston, M., Oligomer Formation Pathways in Secondary Organic Aerosol from MS and MS/MS Measurements with High Mass Accuracy and Resolving Power. *J. Am. Soc. Mass Spectrom.* **2012,** *23*, (6), 1097-1108.

27. Kristensen, K.; Watne, Å. K.; Hammes, J.; Lutz, A.; Petäjä, T.; Hallquist, M.; Bilde, M.; Glasius, M., High-Molecular Weight Dimer Esters Are Major Products in Aerosols from α-Pinene Ozonolysis and the Boreal Forest. **2016,** *3*, (8), 280-285.

28. Zhao, R.; Kenseth, C. M.; Huang, Y.; Dalleska, N. F.; Kuang, X. M.; Chen, J.; Paulson, S. E.; Seinfeld, J. H., Rapid Aqueous-Phase Hydrolysis of Ester Hydroperoxides Arising from Criegee Intermediates and Organic Acids. **2018,** *122*, (23), 5190-5201.

29. Kołodziejczyk, A.; Pyrcz, P.; Błaziak, K.; Pobudkowska, A.; Sarang, K.; Szmigielski, R., Physicochemical Properties of Terebic Acid, MBTCA, Diaterpenylic Acid Acetate, and Pinanediol as Relevant α-Pinene Oxidation Products. **2020,** *5*, (14), 7919-7927.

30. Doussin, J. F.; Monod, A., Structure–activity relationship for the estimation of OH-oxidation rate constants of carbonyl compounds in the aqueous phase. **2013,** *13*, (23), 11625-11641.

31. Monod, A.; Doussin, J. F., Structure-activity relationship for the estimation of OH-oxidation rate constants of aliphatic organic compounds in the aqueous phase: alkanes, alcohols, organic acids and bases. **2008,** *42*, (33), 7611-7622.

32. Lai, C.; Liu, Y.; Ma, J.; Ma, Q.; Chu, B.; He, H., Heterogeneous Kinetics of cis-Pinonic Acid with Hydroxyl Radical under Different Environmental Conditions. **2015,** *119*, (25), 6583-6593.

33. Tan, Y.; Perri, M. J.; Seitzinger, S. P.; Turpin, B. J., Effects of Precursor Concentration and Acidic Sulfate in Aqueous Glyoxal−OH Radical Oxidation and Implications for Secondary Organic Aerosol. **2009,** *43*, (21), 8105-8112.

34. ChemAxon Marvin v20.1.0 developed by ChemAxon was used to calculate pK_a of carboxylic acids Website: https://chemaxon.com/products/marvin, Date last accessed: March 16, 2023.

35. Kołodziejczyk, A.; Pyrcz, P.; Pobudkowska, A.; Błaziak, K.; Szmigielski, R., Physicochemical Properties of Pinic, Pinonic, Norpinic, and Norpinonic Acids as Relevant α-Pinene Oxidation Products. **2019,** *123*, (39), 8261-8267.

36. Howell, H.; Fisher, G. S., The Dissociation Constants of Some of the Terpene Acids. **1958,** *80*, (23), 6316-6319.
